# Systemic Immunity Triggered by Boron Neutron Capture Therapy via Manganese-Borate Complex

**DOI:** 10.1016/j.mtbio.2026.102904

**Published:** 2026-02-05

**Authors:** Chang Chen, Chao Wang, Lin Zhang, Yue Yu, Xu Li, Wenhao Pan, Yuanyu Liu, Jiheng Wang, Maosong Yang, Bing Hong, Xingguang Hu, Jichao Wang, Yuzhong Qian, Xiancai Meng, Yinghuai Zhu, Zhigang Liu, Shaobo Huang, Lizheng Liang, Jinhui Wu, Yang Liu

**Affiliations:** a Institute of Energy, Hefei Comprehensive National Science Center (Anhui Energy Laboratory), Hefei, Anhui, 230031, China; b Department of General Surgery, Affiliated Drum Tower Hospital, Medical School of Nanjing University, Nanjing, 210008, China; c Cancer Center, Dongguan Key Laboratory of Precision Diagnosis and Treatment for Tumors, Guangdong Engineering Research Center of Boron Neutron Therapy and Application in Malignant Tumors, The Tenth Affiliated Hospital, Southern Medical University (Dongguan People's Hospital), Dongguan, 523059, China; d Medical 3D Printing Center, Orthopaedic Institute, Department of Orthopaedic Surgery, The First Affiliated Hospital, School of Biology and Basic Medical Sciences, Suzhou Medical College, Soochow University, Suzhou, 215006, China; e State key Laboratory of Precision and Intelligent Chemistry, Department of Polymer Science and Engineering, School of Chemistry and Materials Science, University of Science and Technology of China, Hefei, 230026, China; f University of Science and Technology of China, Huainan, 232001, China; g Sunshine Lake Pharma Co. Ltd., Dongguan, 523871, China

**Keywords:** Radiotherapy, Boron neutron capture therapy, Immunotherapy, Biomineralized, Albumin, Energy Starvation.

## Abstract

Conventional radiotherapy compromises antitumor immunity through collateral damage to immune cells. While boron neutron capture therapy (BNCT) enables tumor-selective ^10^B (n, α) ^7^Li reactions, clinical agents like boronophenylalanine (BPA) suffer from excessive dosing and exhibit immune-metabolic inertia. We report a biomineralized albumin-based BNCT agent (Albumin@MnB) synthesized from clinically accessible borax, manganese, and albumin, which unlocks neutron capture-triggered immunotherapeutic activation. Albumin@MnB achieves potent tumor suppression at reduced boron doses, demonstrating superior efficacy compared with BPA. Importantly, Albumin@MnB enhances intratumoral immune cell infiltration and suppresses distant tumor growth, synergizing with adoptive T cell immunotherapy and immune checkpoint inhibitors. By integrating tumor-specific radiolytic energy deposition with metabolic reprogramming and immune activation, this strategy establishes boron neutron capture immunotherapy (BNCI) as a multimodal therapeutic paradigm that bridges targeted radiolysis with systemic antitumor immunity.

## Graphical abstract

**Figure:**
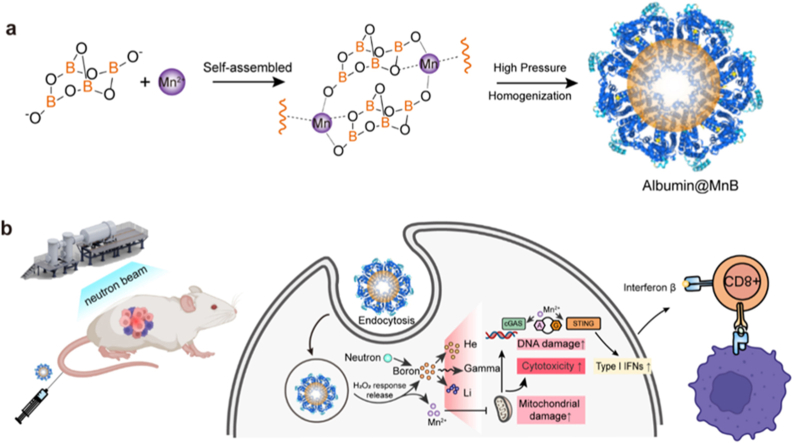

## 1 Introduction

Radiotherapy (RT) has gained recognition as a dual-edged strategy for modulating the immunosuppressive tumor microenvironment (TME) [[1], [2], [3]]- while capable of reversing immune evasion mechanism through immunogenic cell death and tumor-associated antigen release, conventional photon/particle irradiation inevitably damages circulating and tumor-infiltrating immune cell [1,4,5]. Neutron capture therapy (NCT), as an interdisciplinary targeted cancer therapy that complements conventional treatments, has its core branch—boron neutron capture therapy (BNCT) [6]—a precision radiotherapy leveraging thermal neutron-induced nuclear reactions in ^10^B-laden cells, which enables spatially selective killing of tumor cells with minimal damage to normal tissues [[7], [8], [9], [10]](the dual threshold of “fluence ≥ 1×10^12^ n cm^-2^ plus tumor ^10^B ≥ 20 μg g^-1^” [11]). The central value of boron-based compounds in BNCT drug design, drug delivery, and molecular diagnostics has been systematically reviewed and corroborated by recent studies; their fundamental investigation as hydrophobic pharmacophores or boron-cluster carriers has established an essential theoretical and practical framework for the advancement of targeted radiotherapy [[12], [13], [14]]. Nevertheless, current BNCT protocols still rely on supra-pharmacological doses (∼500 mg/kg of L-boronophenylalanine (BPA)) have yet to demonstrate clinically meaningful immune activation against malignancies, highlighting the urgent need for next-generation agent for boron neutron capture immunotherapy (BNCI) [[15], [16], [17], [18], [19], [20], [21]].

**Fig. 1:**
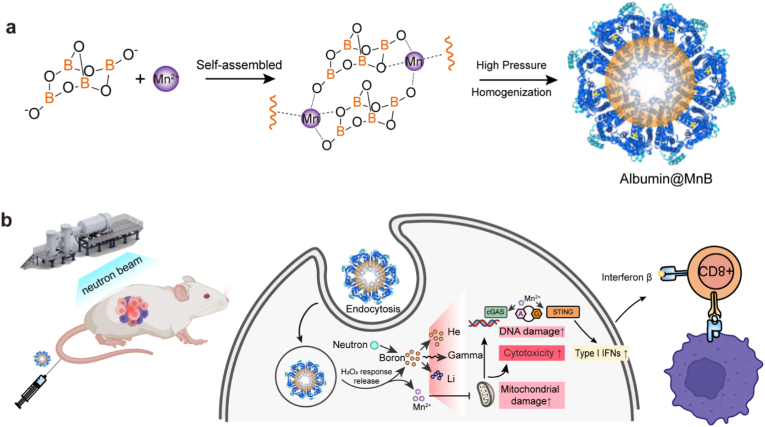
Scheme of Manganese borate nanoparticles for boron neutron capture immunotherapy. Albumin@MnB induces potent localized cytotoxicity via neutron capture and triggers systemic anti-tumor immunity. This multimodal platform, bridging precision radiobiology with immunotherapy, demonstrates the feasibility of boron neutron capture immunotherapy (BNCI).

Building upon recent advancements in BPA delivery optimization that have propelled BNCT progress [[22], [23], [24], [25], [26]], critical limitations of historical agents demand attention [27]. While borax—the pioneering BNCT compound—boasts a 25.6 wt% boron density (3.7-fold higher than BPA's 4.81 wt%) and superior cost-effectiveness [28], its clinical discontinuation resulted from inadequate tumor cell targeting specificity [29,30]. Notably, relevant research has reported that transition metal ions can modulate the tumor microenvironment and enhance the efficacy of targeted radiotherapy by regulating intracellular redox homeostasis [31]. Moreover, mechanochemical synthesis has been utilized to construct boron-based nanobioconjugates via metal alloying, further validating that integrating transition metals with boron materials is a feasible strategy to optimize targeted radiotherapy [32]. Meanwhile, nano-delivery systems are regarded as an ideal strategy to overcome the targeting shortcomings of conventional boron agents, enabling tumor-selective accumulation while maintaining high boron payloads [33]. As a nanodelivery carrier, albumin boasts distinct advantages. As the most abundant endogenous protein in human plasma, it exhibits excellent biocompatibility, with a single clinical infusion dose of up to 20–50 grams, and currently underpins the application of over ten marketed drugs. It passively targets tumors via the enhanced permeability and retention (EPR) effect, while the neonatal Fc receptor (FcRn) recycling pathway extends its half-life to 21 days, thus integrating the merits of tumor targeting and sustained release. Studies from our group and other teams have demonstrated that metal ions exhibit remarkable immunotherapeutic potential [[34], [35], [36], [37], [38], [39], [40]], while the oxygen-rich coordination matrix of borax enables spontaneous self-assembly with transition metals through dative bonding. The co-delivery of two or even multiple drugs can be achieved using nano-delivery system, an emerging and promising technology that has been well-documented in BNCT research for optimizing boron targeting and payload efficiency. These synergistic findings inspire our development of metal-borax hybrid nanoplatforms featuring ultrahigh boron payloads for BNCI. (Fig. 1).

In the study, we present a manganese borate-engineered albumin (Albumin@MnB) strategy. Our screening revealed that manganese ions significantly enhance BNCT-induced cytotoxicity. Notably, the manganese-borate complex demonstrates H_2_O_2_-responsive degradation characteristics. Albumin@MnB exhibits tumor-targeting capabilities comparable to BPA while simultaneously serving as an MRI contrast agent. Crucially, Albumin@MnB demonstrates superior antitumor efficacy compared to BPA, and elicits unique immunostimulatory effects, leading to significant enhancement of T-cell infiltration into tumor tissues and induction of abscopal effects. When integrated with immune checkpoint inhibitors and adoptive T-cell immunotherapy, Albumin@MnB exhibits potent tumor suppressive effects while also effectively inhibiting tumor recurrence. This multifunctional nanoplatform establishes a robust foundation for the development of next-generation BNCT.

## 2 Materials and methods

### 2.1 Cell lines

The B16, 4T1, B16-OVA and 3T3 cell lines were acquired from Procell Life Science &Technology, where these lines were authenticated using morphology, karyotyping and PCR-based approaches and tested for mycoplasma. B16 cells, B16-OVA cells and 3T3 cells were cultured in Dulbecco’s Modified Eagle’s Medium (DMEM) (Gibco) supplemented with 10% fetal bovine serum (FBS) (Gibco), 100 U/mL penicillin, 100 μg/mL streptomycin and gentamicin 50 μg/mL and grown in a humidified atmosphere with 5% CO_2_ at 37 °C. The cell cultures were maintained below a 60% confluence. And the early passage cultures (between four and nine) were utilized for the experiments. 4T1 cells were cultured in Roswell Park Memorial Institute 1640 (RPMI 1640) medium, and the other culture conditions of 4T1 cells are the same as mentioned above.

### 2.2 Animals

Mice of the C57BL/6 strain, weighing 18-20 g, were purchased from GemPharmatech. Animals were observed daily in case of any clinically relevant abnormalities during the study period. If any of the mice was moribund due to therapeutic toxicity, severe ulceration around the subcutaneous tumor appeared in mice or the body weight of the mice decreased by 20% compared with the pre-study, the mice would be euthanatized. All the animal experiments were approved by the Institutional Animal Care and Use Committee (IACUC) of University of Science and Technology of China (ethical approval number USTCACUC25020124076).

### 2.3 Preparation and Characterization of Albumin@MnB

Under ice-bath conditions, manganese ions were introduced into the albumin solution followed by stirring at 500 rpm for 10 minutes. Sodium tetraborate solution was then promptly added to the mixture with continued agitation for an additional 10 minutes. The resulting solution was concentrated and subjected to buffer exchange using a 100 kDa ultrafiltration membrane. The concentrated solution was subsequently processed through a high-pressure homogenizer at 4 °C for 30 minutes the process was carried out at 4 °C, the homogenization pressure was set at 600 bar, and the number of homogenization cycles was 30), ultimately yielding the Albumin@MnB solution.

The transmission electron microscopy (TEM) images were taken on a JEOL JEM-2200FS microscope. Energy dispersive spectroscopy (EDS, Thermo Fischer, FEI Talos F200S, Super-X) spectra were obtained to confirm the chemical element composition. And the particle size and zeta potential of Albumin@MnB and MnB were subjected to dynamic light scattering (DLS) analysis.

### 2.4 Antitumor activity in vitro

#### 2.4.1 CCK-8 assay

The effect of metal ions on the cell viability of B16 cells treated with BNCT were determined by CCK-8 experiment. B16 cells were seeded in a 96-well plate (5×10^3^ cells/well). B16 cells were treated with different metal ions (including Ca^2+^, Mg^2+^, Zn^2+^, Cu^2+^, Fe^3+^, and Mn^2+^) at diferent concentrations (0, 5, 10, 20, 40, 80, and 160 μM) for 24 h. Six replicates were used for each concentration of different metal ions, and the same volume of blank solvent was used as a control. Then, the medium was removed, and 100 μL of DMEM containing 10% CCK-8 reagent was added to each cell well, and after incubation for 1 h, optical density (OD) was determined by an automatic enzyme labeler (450 nm).

#### 2.4.2 Flow cytometry

The effects of BNCT and different metal ion treatments on apoptosis of B16 cells were detected by flow cytometry (CytoFlex, Beckman). Cells are harvested and washed with cold PBS, then resuspended in 1 × Binding Buffer to a concentration of approximately 1×10^6^ cells/mL. The cell death mechanism was investigated by an Annexin V-FITC/PI Apoptosis Detection Kit (Beyotime) according to the manufactu rer’s instructions. Annexin V-FITC and PI staining solutions are added, and the mixture is incubated at room temperature in the dark for 20 minutes. Subsequently, 1 × Binding Buffer is added to bring the total volume to 400 μL. The sample is gently mixed and immediately (within 1 h) analyzed using the flow cytometer.

#### 2.4.3 Detection of mitochondrial membrane potential

Flow cytometry was used to detect the effects of Mn^2+^ treatment on membrane potential of mitochondrial monomers and polymer of B16 cells. Initially, cells are collected and washed twice with 1 × PBS, then resuspended in cell culture medium to a concentration of approximately 1×10^6^ cells/mL. Subsequently, JC-1 dye (100 × ) is added to achieve a final concentration of around 10 μM, and the mixture is incubated at 37 °C for 15-30 min. After incubation, cells are washed twice with pre-cooled 1 × PBS to remove unbound dye. Finally, cells are resuspended in 500 μL of pre-cooled 1 × PBS and immediately analyzed using the flow cytometer.

#### 2.4.4 ICP-MS quantification of the release of Albumin@MnB in vitro and in vivo

(1) The release of ^10^B

To determine the release of ^10^B from Albumin@MnB in vitro, B16 cells were first treated with Albumin@MnB and then exposed to H_2_O and various concentrations of H_2_O_2_ for 5 min, 15 min, 30 min, 60 min, 2 h. Cells were collected, centrifuged to obtain the supernatant, and the concentration of ^10^B in each sample was measured. In vivo, Albumin@MnB was injected intratumorally into B16 tumor-bearing mice. Blood samples were collected at 0, 3, 6, and 24 h post-injection to measure the concentration of ^10^B at each time point, reflecting its circulation in the bloodstream. Tissue samples from the heart, liver, spleen, lung, kidney, and tumor were collected. Approximately 0.05 g of homogenized animal tissue samples were accurately weighed and transferred into 5 mL polytetrafluoroethylene (PTFE) digestion tubes. Then, 0.5 mL of concentrated nitric acid was added to each tube, which was immediately sealed with a matching lid. The sealed digestion tubes were placed in a thermal oven, heated to 85 °C at a ramp rate of 5 °C/min, and incubated at this temperature for 2 hours. After the incubation, the oven was cooled down to room temperature slowly. The digested solutions were transferred into 10 mL polyethylene volumetric flasks and diluted to the calibration mark with ultrapure water. For the inductively coupled plasma mass spectrometry (ICP-MS) analysis, an Agilent 7900 instrument was employed. Specifically, after turning on the condensation and exhaust systems, the instrument’s spray chamber was purged with nitrogen gas at a flow rate of 0.1 L/min for 5 minutes. Subsequently, the flow rates of argon, nitrogen, and oxygen were set to 1, 1, and 0.1 L/min, respectively; the operating temperature was adjusted to 25 °C, and the plasma generator was activated to achieve stable plasma combustion. The standard addition method was selected for boron quantification, with the following parameters: injection time of 30 s, stabilization time of 30 s, and flushing time of 30 s. The concentration range of the standard curve was calibrated to 0.1–100 ppb. Prior to sample analysis, instrument tuning was performed using a 1 ppb standard tuning solution. After the instrument achieved stable performance, sample solutions were introduced for detection. Between consecutive sample injections, the sampling system was rinsed with a mixture of 3% nitric acid and deionized water for 30 s to eliminate cross-contamination. Graphs and statistical data were generated using GraphPad Prism.

(2) The release of Manganese

For the in vitro Mn release assay, the simulated physiological medium was prepared using PBS at pH 7.4, supplemented with 50 μM H_2_O_2_. After thorough mixing, the medium was pre-equilibrated in a constant temperature environment at 37 °C. Albumin@MnB was added to the pre-equilibrated simulated physiological medium described above and incubated in a constant temperature incubator at 37 °C with 5% CO_2_ for in vitro release studies. Supernatant samples from the release system were collected at seven time points: 0, 1, 2, 3 and 5 h post-incubation. The ICP-MS was employed for the quantitative determination of Mn concentration in the supernatant. Three parallel samples were set for each time point, and the average value was used to calculate the cumulative release rate of Mn, so as to analyze its in vitro release kinetic characteristics.

For the in vivo Mn distribution and release assay, the tumor-bearing mice were intravenously injected with Albumin@MnB via the tail vein. At five time points (6, 12, 24, 48, and 72 h post-administration), 3 mice were randomly selected from each group and sacrificed by cervical dislocation. The mice were dissected promptly to isolate tumor tissues and major normal organs (liver, kidney, spleen). The tissue processing procedure was performed in accordance with that described in (1) The release of ^10^B. The ICP-MS was used for the quantitative determination of Mn concentration in the digested solutions. The Mn content per gram of tissue (μg/g) was calculated to analyze the distribution pattern, retention time, and clearance characteristics of Mn in tumor tissues and major normal organs in vivo.

#### 2.4.5 q-PCR

Total RNA was extracted using the TRIZOL reagent method and then reverse transcribed into cDNA using reverse transcription supermix. Quantitative real-time PCR (q-PCR) was conducted using 2 × SYBR qPCR master mix (Q311-02, Vazyme, China). The primers (Table S1) were synthesized by Sangon Biotechnology Co. (China). Relative gene expression was calculated using the 2−ΔΔCT method with β-actin as an endogenous reference.

#### 2.4.6 Characterization of IFN-β and CXCL10 expression

B16 cells were seeded at a density of 2 × 10^5^ cells/mL in 6-well culture plates and treated with B, Mn, and Albumin@MnB, respectively. After 24 h, the cells were collected and centrifuged. The concentrations of INF-β and CXCL10 in the samples were measured using mouse INF-β ELISA kits and mouse CXCL10 ELISA kits, respectively.

#### 2.4.7 Transcriptome analysis

Initially, high-quality total RNA was extracted from each sample group and subjected to RNA quality assessment. Subsequently, cDNA libraries were constructed from the qualified RNA samples. These libraries were then sequenced using a high-throughput sequencing platform to generate a substantial amount of transcriptomic sequence data. During the data analysis phase, differential expression analysis was performed using the DESeq2 software package. Genes with an adjusted P value less than 0.05 and an absolute log2 fold change greater than 1 were identified as differentially expressed genes. Additionally, visualization tools such as volcano plots and box plots were employed to graphically represent the differentially expressed genes.

#### 2.4.8 Metabolomics analysis

Collect and count the cells from each group, taking 1×10^7^ cells per group. Add extraction solution (methanol: acetonitrile: water=2:2:1, v/v)and vortex mix for 30 seconds; disrupt the cells using a cell disruptor for 15 minutes; let the samples stand at -40 °C for 1 hour; then centrifuge at 4 °C at 12,000 rpm for 15 minutes; transfer the supernatant to a sample vial for analysis. Chromatographic separation is performed using a Vanquish Ultra-High Performance Liquid Chromatography (UHPLC) system (Thermo Fisher Scientific) with a Waters ACQUITY UPLC BEH Amide column (2.1 mm × 50 mm, 1.7 μm). For the mobile phase of the liquid chromatography: Phase A was an aqueous phase containing 25 mmol/L ammonium acetate and 25 mmol/L ammonia water, while Phase B was acetonitrile. The sample tray temperature was set at 4 °C, and the injection volume was 2 μL. The detailed parameters of the Orbitrap Exploris 120 mass spectrometer are as follows: Sheath gas flow rate: 50 Arb; Aux gas flow rate: 15 Arb; Capillary temperature: 320 °C; Full MS resolution: 60000; MS/MS resolution: 15000; Collision energy: SNCE 20/30/40; Spray Voltage: 3.8 kV (positive mode) or -3.4 kV (negative mode). The raw data are preprocessed, including filtering outliers, handling missing values, imputation, and normalization. Principal component analysis (PCA) and orthogonal projections to latent structures discriminant analysis (OPLS-DA) are conducted using SIMCA software (V18.0.1, Sartorius Stedim Data Analytics AB, Umea, Sweden). The criteria for identifying differential metabolites are P<0.05 and VIP>1.

### 2.5 Antitumor activity in vivo

#### 2.5.1 Tumor-volume measurements

The perpendicular diameters of the tumor were measured using a caliper, and the tumor volume was estimated using the formula V=0.5 × A × B^2^, where A denotes the longer diameter and B represents the shorter diameter. Tumor volume was assessed every other day. Statistical differences in the average tumor growth curves were analyzed using two-way ANOVA with Bonferroni correction, considering time and volume as variables.

#### 2.5.2 Detection of immune cells in tumors

14 days after neutron beam irradiation, the tumors from both sides were excised and minced into small pieces in 2 mL of DMEM medium. Subsequently, the samples were subjected to digestion in 2 mL of DMEM containing 500 μg/mL collagenase IV (Invitrogen), 100 μg/mL hyaluronidase, and 20 U/mL DNase (Macklin) at 37 °C for 30 min. After digestion, the reaction was terminated by adding DMEM medium supplemented with FBS, and red blood cells were lysed using ACK Lysis Buffer (Life Technologies). The digested suspensions were then filtered through a 300-mesh cell strainer and washed three times with PBS. The resulting single-cell suspensions were stained with Zombie-UV viability dye and antibodies against mouse CD45, CD3, CD8, and CD11c for 30 minutes on ice. Following staining, the cells were washed with FACS buffer and analyzed by flow cytometry.

#### 2.5.3 MRI characterization

Given that divalent manganese inherently possesses a magnetic resonance imaging (MRI) signal, the metabolism of Mn^2+^ in mice can be distinctly tracked using MRI following subcutaneous injection of Albumin@MnB. In the experiment, mice were administered Albumin@MnB via subcutaneous injection, then anesthetized and immobilized. MRI scans were conducted at 0, 3 and 24 h post-injection to meticulously observe the metabolic alterations of Mn^2+^ within the mice. All imaging was carried out on a 9.4 T/400 mm wide-bore system (Agilent Technologies, Santa Clara, CA, USA) equipped with a volume RF coil. For in-vitro phantoms and live-cell samples, T1 was determined with a rapid acquisition relaxation-enhanced sequence: TE fixed at 16.18 ms; TRs stepped through 843, 1 117, 1 141, 1 836, 2 343, 3 050, 4 233 and 9 000 ms; FOV 32 mm × 32 mm, matrix 128 × 128, bandwidth 50 kHz, five slices, slice thickness 1 mm. T1-weighted images were reconstructed from the TR = 843 ms dataset.

#### 2.5.4 Living imaging

Tumor-bearing mice constructed with B16 and 4T1 cells were employed to evaluate the anti-tumor efficiency of Albumin@MnB. Stable firefly luciferase-expressing 4T1 and B16 cell lines were generated via lentiviral transduction and subcutaneously inoculated into BALB/c mice and C57BL/6 mice, respectively. The bioluminescence imaging using an IVIS imaging system after intraperitoneal injection of 200 μL of luciferin substrate (150 mg/kg).

#### 2.5.5 BNCT treatments

An accelerator-driven neutron source based on a D–D neutron generator, developed by the Institute of Energy at the Hefei Comprehensive National Science Center, was used to irradiate tumor cells and mouse models. Fast 180 keV neutrons produced via the D–D reaction were thermalized by a precisely machined polyethylene assembly to deliver the required thermal-neutron beam. During exposure, awake mice were immobilized in a custom-built, non-anesthetic fixation device (Fig. S1) mounted at the irradiation port; each animal was positioned with its tail aligned to the beam axis so that only the tumor lay within the thermal field while the rest of the body remained outside, ensuring selective targeting. The tumor region and cells received a 4 h irradiation at a thermal-neutron fluence rate of 2 × 10^7^ n cm^-2^ s^-1^. The neutron energy spectrum was dominated by thermal neutrons (energy < 0.5 eV), accompanied by minor fractions of epithermal neutrons (0.5–10 keV, ∼3%) and fast neutrons (>10 keV, <1%). The average neutron flux at the irradiation position was calibrated and measured to be 2.86 × 10^7^ n·cm^-2^·s^-1^, corresponding to a total neutron dose of 4.12 × 10^11^ n·cm^-2^ at the tumor site. The irradiation dose was determined by Monte Carlo simulations of neutron transport, which were based on the geometric parameters of the accelerator-based neutron source and the irradiation field. These simulations were used to calculate the spatial flux distribution across the target volume, which was then converted into corresponding dose values, with the total delivered irradiation dose being ∼15 Gy. Borated polyethylene surrounding the moderator absorbed scattered neutrons, minimizing peripheral background.

### 2.6 Statistical analysis

All data in the present study are expressed as mean ± s.d. unless indicated in the figure legends. The GraphPad Prism (v7.0) was used for statistical analysis. The results were presented as mean ± SD. Student t-test for two group comparisons and one- or two-way ANOVA were employed for multiple group comparisons. Data were normally distributed and the variance between groups was similar. All the values were reported as mean ± s.d. with the indicated sample size. All the animal studies were performed after randomization. In all cases, a P value less than 0.05 was considered significant.

## 3 Results and discussion

### 3.1 Manganese ions can boost Boron Neutron Capture Therapy (BNCT)

Metal ions, particularly transition metals, can catalyze peroxide degradation to generate oxidative free radicals, thereby disrupting intracellular redox homeostasis [41,42]. In traditional high-energy X-ray radiotherapy, metal ions have been shown to significantly enhance programmed cell death in tumor cells. However, the reaction mechanisms of BNCT fundamentally differ from conventional X-ray therapy, and the role of metal ions in BNCT remains unexplored. This knowledge gap prompted us to develop a synergistic activity screening strategy combining low-dose BNCT treatment with subsequent metal ion administration.

**Fig. 2:**
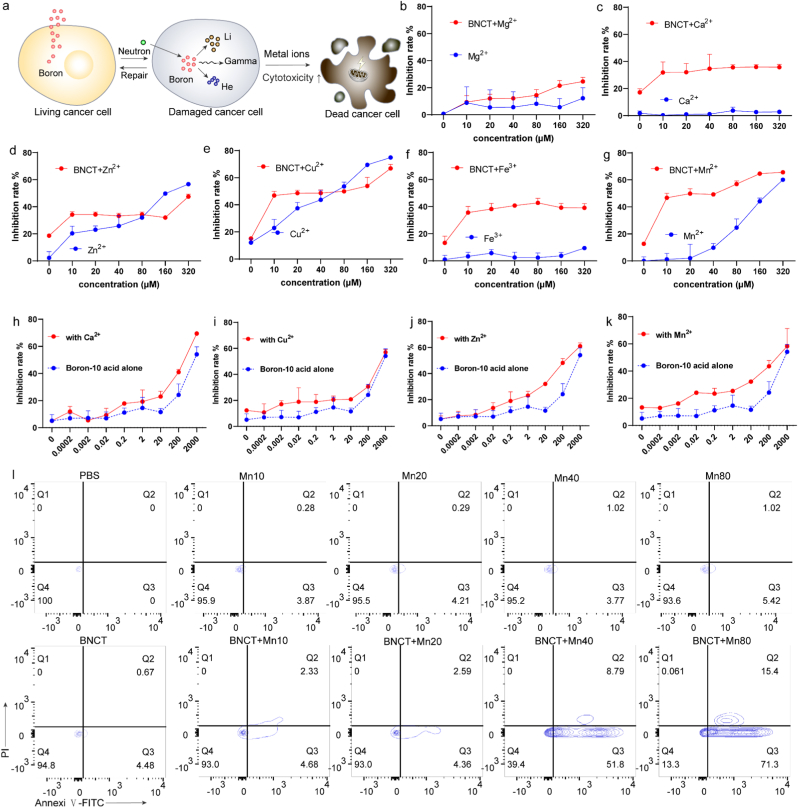
Manganese ions can boost BNCT. a, Mechanism diagram of low dose BNCT and metal ion synergistic therapy. **b-g**, Inhibition rate detection of B16 cells after BNCT alone or in combination with different metal ions including Mg^2+^ (b), Ca^2+^ (c), Zn^2+^ (d), Cu^2+^ (e), Fe^3+^ (f), and Mn^2+^ (g); **h-k**, Boron-10 acid alone or in combination with different metal ions including Ca^2+^ (h), Cu^2+^ (i), Zn^2+^ (j), and Mn^2+^ (k). (n=3, Data represent mean ± s.d.). **l**, Flow cytometry showed that Mn^2+^ could induce BNCT-induced programmed cell apoptosis in a dose-dependent effects.

Systematic screening of transition metals revealed manganese's unique capacity to enhance BNCT efficacy. We established a screening platform combining subtherapeutic BNCT doses (9.25 mM) with subsequent metal ion administration (0-320 μM) to evaluate synergistic effects (Fig. 2a). We found that Mg^2+^(Fig. 2b), Ca^2+^(Fig. 2c), and Fe^3+^(Fig. 2f) could moderately enhance BNCT-induced programmed cell death, but exhibited no significant dose-dependent effects. Even at 320 μM concentrations, these metal combinations failed to reduce cell viability by more than 50% (Fig. 2b, c and f). Cu^2+^ demonstrated inherent cytotoxicity but showed no observable synergistic effects (Fig. 2e). Notably, Zn^2+^ and Mn^2+^ displayed concentration-dependent synergism, with Mn^2+^ achieving significant cell death potentiation (53% viability) at 5 μM >32-fold lower than Zn^2+^’s effective concentration (160 μM) (Fig. 2d, g). At the same time, the administration of metal ions (Ca^2+^, Cu^2+^, Zn^2+^, Mn^2+^) in combination with Boron-10 acid (0-2000 μg/mL) shows that the presence of Ca^2+^ (Fig. 2h), Cu^2+^ (Fig. 2i), Zn^2+^ (Fig. 2j), and Mn^2+^ (Fig. 2k) can all enhance the inhibitory effect of Boron-10 acid, but Mn^2+^ has the most significant enhancement effect on the inhibitory effect of Boron-10 acid. Further analysis using Annexin V/PI apoptosis staining revealed a dose-dependent increase in Annexin V-positive cell populations with ascending manganese concentrations (Fig. 2l).

**Fig. 3:**
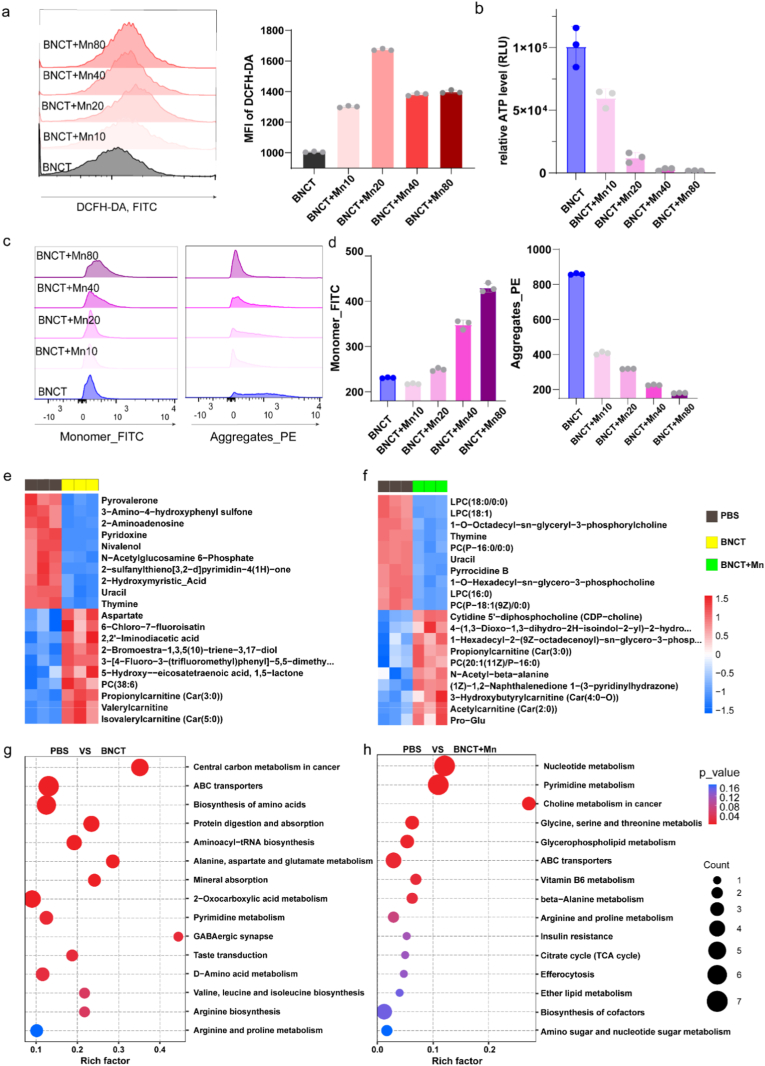
Manganese ions impair mitochondrial function. a, Flow cytometry analysis of the effects of BNCT and different concentrations of Mn^2+^ on B16 cell apoptosis. **b**, Bioluminescent ATP assays of ATP release levels in B16 cells treated with BNCT and different concentrations of Mn^2+^ (n=3, Data represent mean ± s.d.). **c**, Flow cytometry analysis of the effects of BNCT and different concentrations of Mn^2+^ on mitochondrial monomer and polymer membrane potential in B16 cells by JC-1 staining. **d**, Quantitative analysis of the fluorescence intensity of mitochondrial monomer and polymer in B16 cells after the BNCT and different concentrations of Mn^2+^ treatments. **e**, Hierarchical clustering analysis heat map of the PBS group vs the BNCT group. **f**, Hierarchical clustering analysis heat map of the PBS group vs the BNCT + Mn group. **g**, KEGG enrichment map of differential metabolites between the PBS group and the BNCT group. **h**, KEGG enrichment map of differential metabolites between the PBS group and the BNCT + Mn group.

To elucidate the mechanism underlying manganese-enhanced boron neutron capture therapy (BNCT), we systematically investigated cellular responses to Mn^2+^ co-treatment. Intracellular reactive oxygen species (ROS) detection using DCFH-DA revealed significant ROS elevation in Mn^2+^-supplemented groups compared to BNCT alone (Fig. 3a). This aligns with the well-established paradigm that nanomaterial or metal ion-assisted radiation therapy amplifies oxidative stress to enhance anti-tumor efficacy—similar to how carbon nanoparticles boost proton therapy effectiveness by increasing high-LET secondary particle doses and disrupting intracellular redox homeostasis [43]. Concurrently, we observed a marked reduction in cellular ATP levels (Fig. 3b), prompting investigation of mitochondrial functionality. JC-1 staining demonstrated significant mitochondrial membrane potential collapse in co-treated cells (Fig. 3c, d), indicating mitochondrial impairment. Subsequent metabolomic analysis revealed distinct metabolic profile alterations following Mn^2+^ administration, particularly showing substantial depletion of phosphatidylcholine homeostasis - a critical component of cellular membranes (Fig. 3e, f). Pathway enrichment analysis further identified significant perturbations in choline metabolism, which is important for maintaining the stability of membranes such as mitochondria (Fig. 3g, h).

Collectively, our findings establish Mn^2+^ as a potent BNCT synergizer functioning at through mitochondrial-associated damage pathways, primarily disrupting cellular membrane homeostasis. This mechanistic insight advances optimization strategies for BNCT through rational metal ion co-administration, highlighting membrane integrity modulation as a promising therapeutic enhancement approach.

### 3.2 Assembly and hydrogen peroxide-responsive release of manganese borate nanoparticle

While L-boronophenylalanine (BPA) achieves tumor targeting through LAT1-mediated transport, its limited boron content (4.81 wt%) necessitates high-dose administration (∼500 mg/kg), imposing significant hepatic/renal burdens and systemic toxicity risks including hypotension. This contrasts with early BNCT agents like boric acid derivatives (25.6 wt% boron) that lack targeting specificity but possess superior boron payloads. Previous studies on boron-based radiation therapy have emphasized that the efficacy of boron-dependent therapeutic strategies (whether proton-boron fusion therapy or neutron capture therapy) is fundamentally determined by the selective accumulation of boron in tumor tissues and the efficiency of boron delivery systems [44]—an issue that has driven the development of diverse nano-carrier strategies, including boron-cluster conjugates and nanostructured delivery platforms [45]. We therefore set out to construct a delivery system capable of co-delivering transition metals and boric acid while enabling dose sparing and minimal side effects. Using nano-delivery systems to achieve co-delivery of two or even multiple agents is an emerging and well-validated technology in BNCT research for optimizing boron targeting and payload efficiency. We capitalizing on boric acid's oxygen-rich coordination environment, to explore metal borate complexes as high-density boron carriers.

**Fig. 4:**
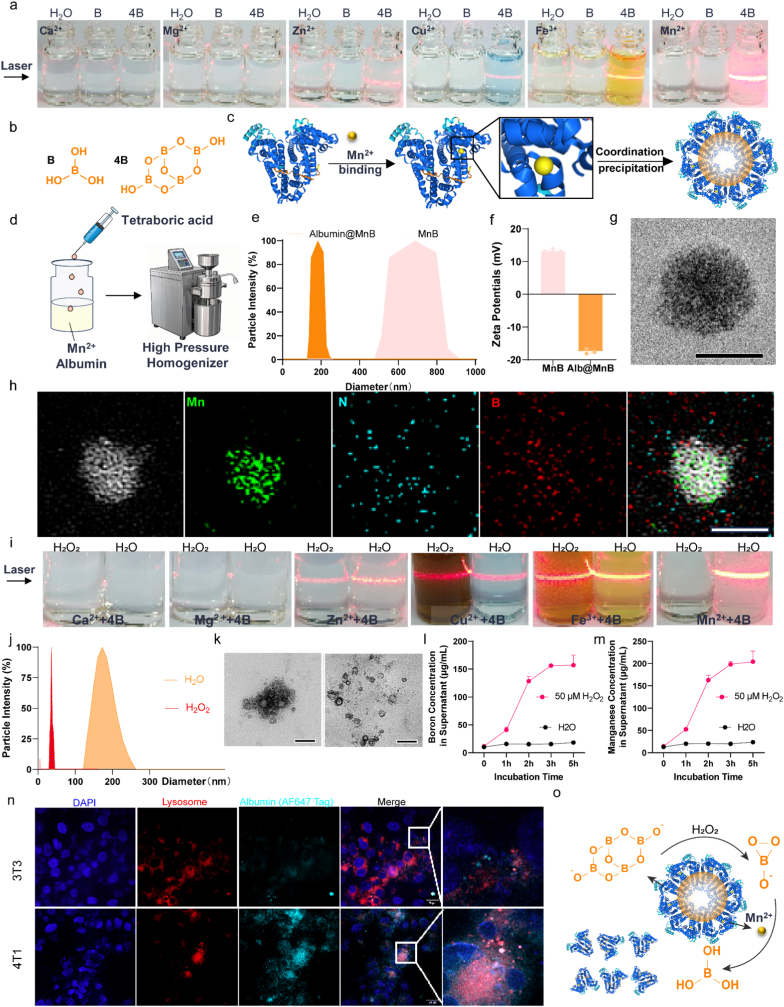
Assembly and hydrogen peroxide-responsive release of Albumin@MnB. a, Self-assembly after coordination with different metal ions with B and 4B. **b**, The structural formula for B and 4B. **c**, Scheme of coordination self-assembly of Mn^2+^ and albumin. **d**, Schematic illustration of the assembly principle between albumin and 4B. **e**, Particle size analysis of Albumin@MnB and MnB. **f**, Zeta potential analysis of Albumin@MnB and MnB. **g**, Transmission electron microscopy (TEM) image of Albumin@MnB. **h**, Elemental distribution in this protein formulation (Scale bars, 200 nm). **i**, Disassembly of different metal ions coordinated with 4B in response to H_2_O_2_. **j**, Particle size analysis of Albumin@MnB under H_2_O and H_2_O_2_ conditions. **k**, TEM images of Albumin@MnB under H_2_O and H_2_O_2_ conditions (Scale bars, 200 nm). **l-m**, Release of boron and Mn from the protein formulation at different H_2_O_2_ concentrations (n=3, Data represent mean ± s.d.). **o**, Laser Scanning Confocal Microscopy (LSCM) images of 4T1 and 3T3 cells (Scale bars, 100 μm). **p**, Schematic illustration of the disassembly principle of the protein formulation under H_2_O_2_ conditions.

Through screening, we discovered that tetraborate mixed with various transition metals in water, including Zn^2+^, Cu^2+^, Fe^3+^ and Mn^2+^, coordinated their self-assembly into coordination polymers with diameters ranging from nanometres to micrometres (Fig. 4a). Other metal ions, such as Ca^2+^ and Mg^2+^, cannot assemble with tetraborate efficiently. In addition, boric acid cannot produce coordination assembly effects with all of the above metal ions (Fig. 4a).

Furthermore, we overcome the spontaneous coordination between tetraborate and transition metals (Zn^2+^, Cu^2+^, Fe^2+^, Mn^2+^), forming metastable assemblies prone to uncontrolled growth (Fig. 4a, b) by employ albumin as a biomimetic scaffold, leveraging its metal-binding domains and emulsifying properties to regulate nanostructure formation (Fig. 4c, d, S2). Consistent with previous reports that bioactive molecules (e.g., vitamin C) can bind to boron to construct stable nanocomposites through coordination interactions [46], albumin leverages its metal-binding domains and emulsifying properties to regulate nanostructure formation. The monodisperse albumin-manganese tetraborate nanocomposites (Albumin@MnB) prepared by this method have very strong stability, and the particle size remains stable after storage at 4 °C for more than 3 months and precisely controlled size (Fig. 4 e-g, S3-4). Dynamic light scattering (DLS) analysis demonstrated that the polydispersity index (PDI) of Albumin@MnB was 0.145 ± 0.006 (n=3). Inductively coupled plasma optical emission spectrometry (ICP-OES) results revealed that the molar ratio of B to Mn in the nanocomposites was 4.03 ± 0.06:1 (n=3). Moreover, the encapsulation efficiency (EE) of albumin, determined via ultracentrifugation (18,000 g, 4 °C, 30 min), reached 69.6% ± 2.01% (n=3). These favorable physicochemical properties are critical for the clinical translation of Albumin@MnB. The elemental distribution map confirmed the uniform co-localization of boron and manganese in the Albumin@MnB nanoparticles (Fig. 4h).

Albumin@MnB exhibited unique tumor microenvironment response characteristics: in the presence of H_2_O_2_, the particle size of Albumin@MnB degraded from 180 nm to less than 20 nm within 30 minutes (Fig. 4j, k). TEM showed that the complete structure of Albumin@MnB was dissociated into dispersed fragments (Fig. 4k). Quantitative analysis of the released boric acid confirmed that Albumin@MnB was dependent on the concentration of H_2_O_2_ [47,48] (Fig. 4l-m and S8). A property we attribute to dual mechanisms: 1) oxidative radical-mediated cleavage of tetraborate ester bonds and 2) Mn^2+^-catalyzed peroxide hydrolysis (Fig. 4n). Cell experiments also further showed that compared with normal 3T3 cells, Albumin@MnB could be more efficiently taken up by 4T1 tumor cells and specifically dissociated in 4T1 tumor cells (Fig. 4o).

### 3.3 MRI tracking and biodistribution of Albumin@MnB

**Fig. 5:**
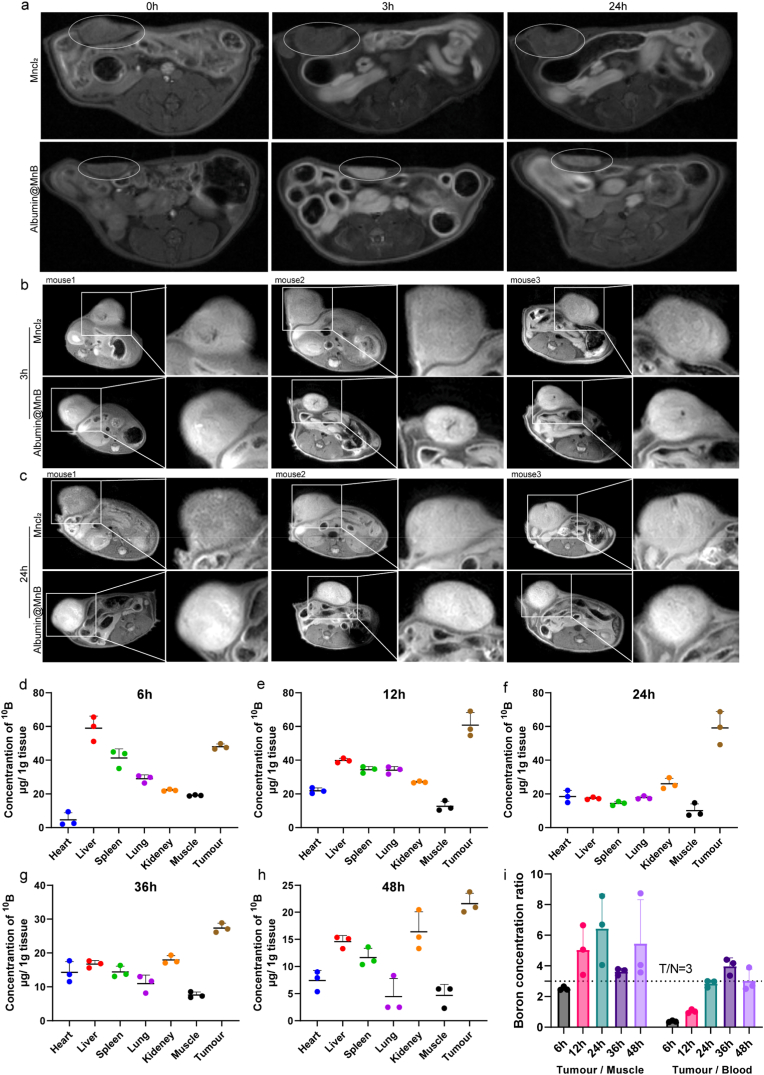
MRI tracking and biodistribution of Albumin@MnB. a, 9.4T MRI imaging showing the distribution of MnCl_2_ and Albumin@MnB in the same mice at 0 h, 3 h, and 24 h post-injection. **b**, 3.0T MRI characterization of Mn^2+^ metabolism at 3 h post-injection of MnCl_2_ or Albumin@MnB with equivalent manganese doses. **c**, MRI characterization of Mn^2+^ metabolism at 12 h post-injection of MnCl_2_ or Albumin@MnB with equivalent manganese doses. d–h, The concentration of boron-10 (^10^B, μg per gram of tissue) in major organs (heart, liver, spleen, lung, kidney, muscle) and tumor tissue of mice at 6 h (d), 12 h (e), 24 h (f), 36 h (g), and 48 h (h) after tail vein injection of Albumin@MnB. i, The tumor-to-muscle (T/N) signal ratios at 6 h, 12 h, 24 h, 36 h, and 48 h after tail vein injection of Albumin@MnB.

The paramagnetic nature of metal ions Mn^2+^ enables real-time MRI monitoring of the distribution and tumor accumulation of Albumin@MnB, thus facilitating precise neutron irradiation guidance. This theranostic integration (imaging + therapy) aligns with the development trend of NCT agents, as reported in previous studies on gadolinium-based compounds with dual MRI contrast and neutron capture functions [49]. Three hours after injection of MnCl_2_ with an equivalent dose of Mn^2+^, mice treated with Albumin@MnB exhibited stronger T1-weighted imaging signals (*r1*= 8.62 mM^−1^⋅s^−1^) in the tumor region compared with mice injected with free MnCl_2_ (Fig. 5a-c). This signal difference became more obvious after 24 hours, indicating that Albumin@MnB gradually released Mn^2+^ in the tumor through the degradation in response to H_2_O_2_ in the tumor microenvironment, producing stronger MRI signals (T1-weighted MRI showed that Albumin@MnB produced a continuously enhanced contrast signal in the tumor area (Fig. 5a-c, S5).

In order to better carry out BNCT treatment based on Albumin@MnB, we further used ICP-MS to quantitatively analyze the boron distribution in mice after Albumin@MnB treatment. Compared with the administration of small-molecule tetraborates containing the same dose of boron, Albumin@MnB significantly prolonged the circulation of boron in the body (Fig. S9a), significantly increased the accumulation of boron in tumors (increased by 4 times), and reduced the accumulation of boron in the kidneys (reduced by more than 40%) (Fig. S9b). We further investigated the biodistribution of the formulation in mice (Fig. 5d–i). Quantitative analysis revealed that the boron concentration in tumors reached 45 μg/g at 24 h post-administration of Albumin@MnB (Fig. 5f), and remained above 20 μg/g by 36 h post-administration (Fig. 5g). Between 24 and 36 h after injection, the tumor-to-muscle and tumor-to-blood boron concentration ratios both exceeded 3 (Fig. 5i), meeting the therapeutic threshold for boron neutron capture therapy (BNCT). In contrast, small-molecule tetraborates alone exhibited no tumor-specific distribution profile. Analogous tumor accumulation of boron and other payloads mediated by nanoparticulate formulations [50] and biomacromolecular carriers [51] has also been documented at 24 h post-administration or beyond. Furthermore, inductively coupled plasma mass spectrometry (ICP-MS) was employed to quantify Mn^2+^ concentrations in tumor tissues and major normal organs (Heart, Liver, Spleen, Lung, Kidney, and Muscle) at multiple time points (12, 24, 36, and 48 h post-injection). These findings verified the sustained in vivo release of Mn^2+^ from the nanoparticles: tumor Mn^2+^ concentrations remained stable at approximately 15 μg/g over a 72-h period (Fig. S9), a profile that secures persistent immunomodulatory activity to synergize with BNCT.

In conclusion, Albumin@MnB achieves the selective accumulation of boron and Mn in the tumor by prolonging the circulation of circulating boron in the body and further realizing the selective accumulation of boron and Mn in the tumor through the degradation and release mechanism in response to the H_2_O_2_ microenvironment of the tumor, thereby realizing MRI-guided patient stratification and neutron irradiation planning for BNCT. At the same time, Albumin@MnB significantly reduces the dosage of small-molecule tetraborates and improves the distribution of boron in the tumor, meeting the ^10^B treatment threshold of BNCT.

### 3.4 Albumin@MnB demonstrates potent antitumor efficacy with immune activation

**Fig. 6:**
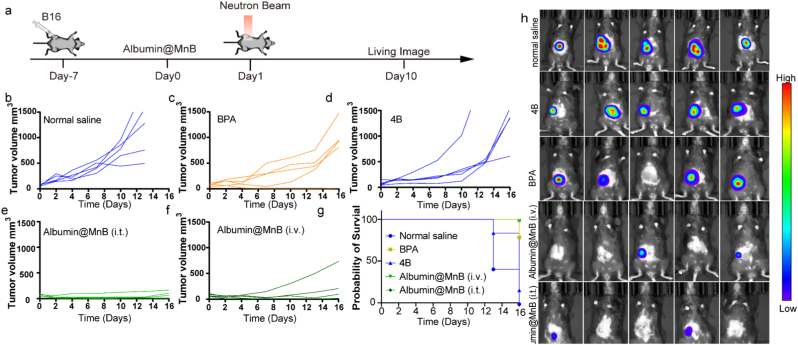
Albumin@MnB demonstrates potent antitumor efficacy with immune activation. a, Experimental timeline. Mice were inoculated with B16 cells 7 days before the start of the experiment, administered boron-equivalent doses (10 mg/kg) of Albumin@MnB on Day 0. 4B and BPA were injected 2 h before irradiation. Neutron beam irradiation on Day 1. Living imaging was performed on Day 10. **b-g**, The impact of different treatments on tumor volume and survival probability. (b) Normal saline group; (c) BPA treatment group; (d) 4B treatment group; (e) Intravenous injection of Albumin@MnB nanoparticles group; (f) Intravenous injection of Albumin@MnB nanoparticles group; (g) Survival probability curves for all groups. (n=5, Data represent mean ± s.d.). **h**, Living imaging results after treatment with normal saline, 4B, BPA, Albumin@MnB (i.v.) and Albumin@MnB (i.t.). The color coding indicates signal intensity, ranging from low (blue) to high (red).

Encouraged by biodistribution data, we further evaluated the therapeutic effect of Albumin@MnB in synergistic with neutron irradiation in vivo. Initial evaluation using the B16 melanoma model (Fig. 6a), which exhibits strong L-boronophenylalanine (BPA) uptake, revealed significant therapeutic advantages. Both tumor growth curves (Fig. 6b-f) and bioluminescence signals (Fig. 6h) demonstrated that a single intravenous injection of Albumin@MnB (10 mg/kg boron equivalent) achieved dose-dependent therapeutic efficacy. Complete tumor eradication was observed in 1/5 mice receiving intravenous administration and 2/5 mice with intratumoral injection (Fig. 6e, f).

**Fig. 7:**
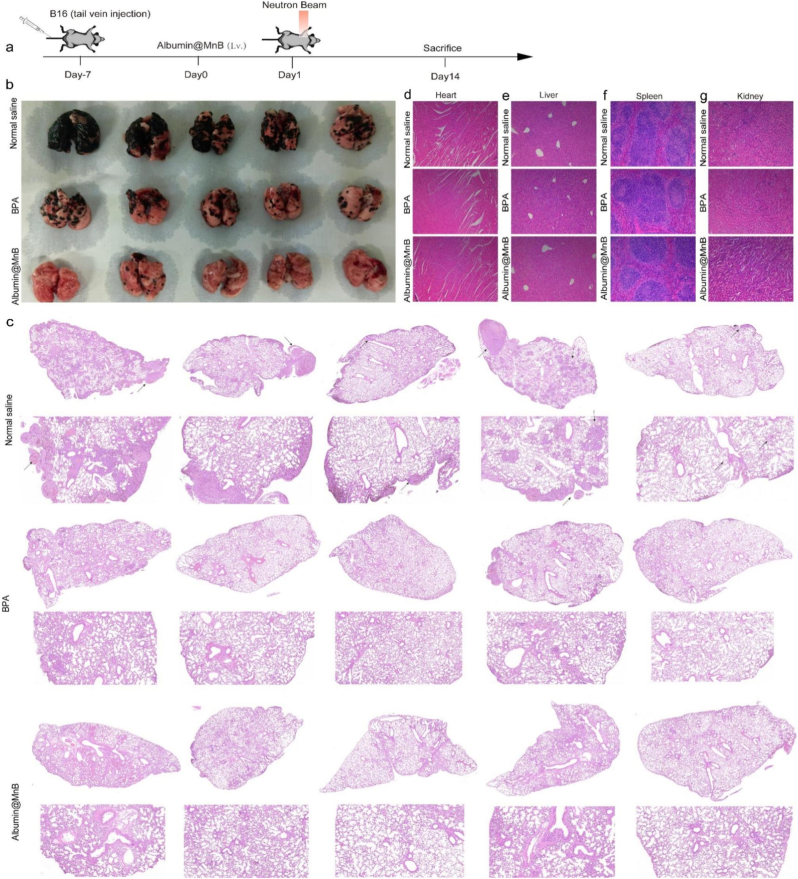
Albumin@MnB mediated potent antitumor efficacy in a mouse melanoma lung metastasis model. a, Experimental timeline. Mice were inoculated with B16 cells 7 days before the start of the experiment. On Day 0, mice received normal saline and Albumin@MnB treatments via intravenous injection (i.v.). BPA were injected 2 h before irradiation. On Day 1, mice were subjected to neutron beam irradiation, and were sacrificed on Day 14 for tissue extraction and sectioning. **b**, Photographs of lung tissue after treatment with saline, BPA, and Albumin@MnB. **c**, Histological sections of tumor tissues from normal saline, BPA and Albumin@MnB treatment groups. **d-g**, H&E-stained sections of heart (d), liver (e), spleen (f), and kidney (g).

**Fig. 8:**
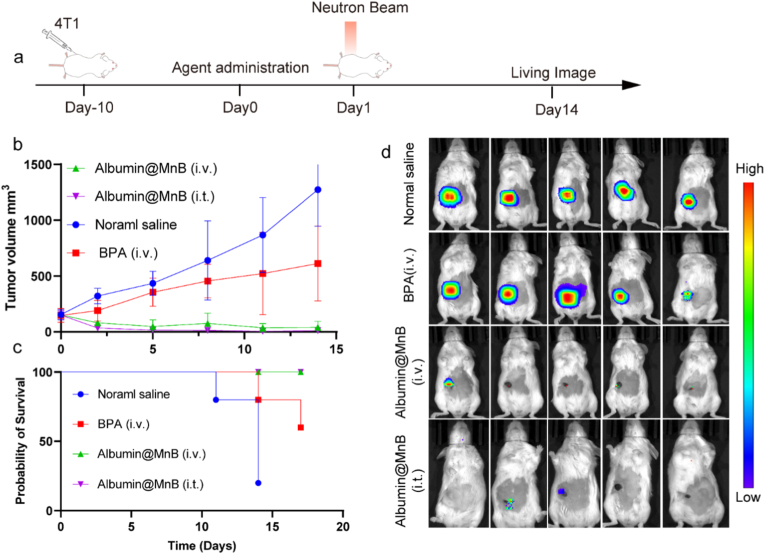
Albumin@MnB mediated potent antitumor efficacy in a mouse breast cancer model. a, Experimental timeline. Ten days before the start of the experiment (Day-10), mice were injected with 4T1 cells. On the day of the experiment (Day 0), mice were injected with saline and Albumin@MnB. BPA were injected 2 h before irradiation. Subsequently, on Day 1, mice were exposed to neutron beam irradiation. Imaging was conducted on Day 10. **b**, Tumor growth curves in mice (n=5, Data represent mean ± s.d.). **c**, Survival probability of mice in different treatment groups. **d**, Living imaging results after different treatment. The color coding indicates signal intensity, ranging from low (blue) to high (red).

To address metastatic challenges, we established an aggressive B16 metastasis model (Fig. 7a). Encouragingly, Albumin@MnB-mediated BNCT significantly eliminated pulmonary metastatic foci (Fig. 7b, c), while showing no apparent histopathological toxicity in major organs (Fig. 7d-g). Furthermore, our assays demonstrated that serum biomarkers associated with hepatic and renal function exhibited no significant differences (Fig. S10).We subsequently evaluated the platform in triple-negative breast cancer (4T1) models with suboptimal BPA uptake (Fig. 8a). Unlike BPA, Albumin@MnB demonstrated potent antitumor activity in both tumor growth curves (Fig. 8b) and bioluminescence monitoring (Fig. 8d), with higher mouse survival rates (Fig. 8c), and achieving complete tumor clearance in 3/5 mice across both administration routes.

These findings collectively demonstrate that Albumin@MnB achieves superior therapeutic efficacy to BPA in both primary and metastatic tumor treatment, while simultaneously suppressing growth in untreated distal tumors, suggesting potent immune activation mediated by this novel agent.

### 3.5 Albumin@MnB-based BNCT boosts immune activation and curbs cellular repair

**Fig. 9:**
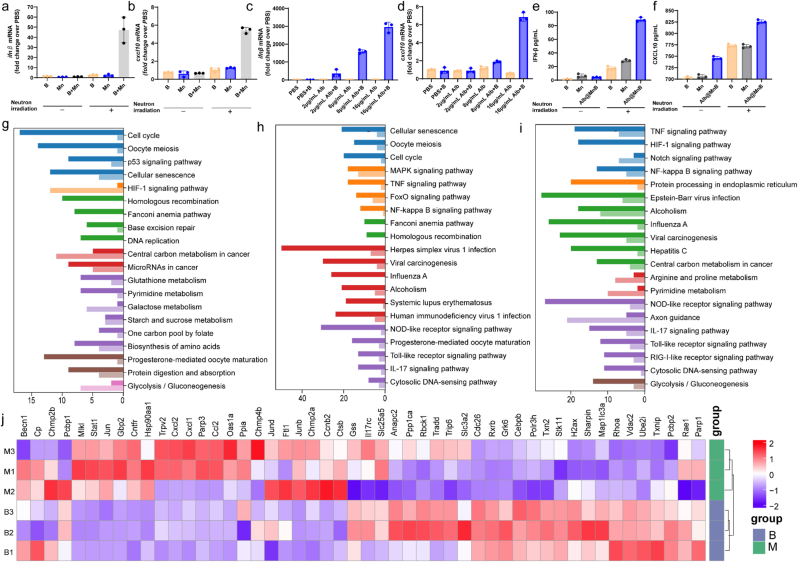
Albumin@MnB boosts immune activation and curbs cellular repair. a-b, q Quantitative PCR assay of ifnβ (a) and CXCL10 (b) in the group of B, Mn and B + Mn. **c-d**, Quantitative PCR analysis of the effects of Albumin@MnB treatment on ifnβ (c) and CXCL10 (d) mRNA. Data represent mean ± s.d. **e-f**, Enzyme-linked immunosorbent assay (ELISA) analysis of IFNβ (e), CXCL10 (f) concentration in tumor supernatants. Data represent mean ± s.d. **g-i**, The Kyoto Encyclopedia of Genes and Genomes (KEGG) pathway enrichment analysis of the DEGs between B and NC group (g), Albumin@MnB and NC group (h), and B and Albumin@MnB group (i). **j**, Enrichment of differentially expressed genes. Data compared by two-way ANOVA. ∗*P* < 0.05, ∗∗*P* < 0.01, ∗∗∗*P* < 0.001.

Our previous study found that radiotherapy-induced damaged DNA leaked into the cytoplasm, and in the presence of Mn, it could significantly activate the anti-tumor immune response induced by the cGAS-STING pathway [24]. Here, we further explored whether BNCT therapy based on Albumin@MnB is related to the activation of the innate immune signal cGAS-STING pathway by Mn. Activation of the cGAS-STING pathway induces upregulation of the expression of interferon-stimulated genes such as IFN-β. Here, we used real-time quantitative PCR to detect the expression of IFN-β and CXCL10, and found that Mn and 4B synergistically induced the expression of IFN-β and CXCL10, which was dependent on neutron radiation (Fig. 9a-b). It was further found that the higher the dose of Albumin@MnB, the more obvious the upregulation of IFN-β and CXCL10 induced, which was positively correlated with the dose (Fig. 9c-d). The protein expression levels of IFN-β and CXCL10 detected by ELISA were consistent with the results of real-time quantitative PCR (Fig. 9e-f). This indicates that BNCT therapy based on Albumin@MnB induces anti-tumor immunity by activating and amplifying DNA damage by Mn.

Transcriptomic profiling uncovered stark mechanistic contrasts: conventional BNCT (Fig. 9g) predominantly activated pro-survival pathways (e.g., DNA replication, homologous recombination), whereas Albumin@MnB-based BNCT shifted the transcriptomic landscape toward innate immunity (Fig. 9h-i). Key immune pathways—including TNF/NF-κB signaling, antigen processing, and interferon response—were robustly upregulated. Concurrently, DNA repair genes (*PARP1*, *XRCC1*) and oxidative stress mitigators (*TXNIP*) were downregulated, suggesting impaired damage resolution (Fig. 9j).

These findings establish Albumin@MnB-based BNCT demonstrates superior innate immune activation and attenuated cellular repair capacity compared to conventional approaches.

### 3.6 Synergistic antitumor efficacy of Albumin@MnB-based BNCT with T-Cell immunotherapy

**Fig. 10:**
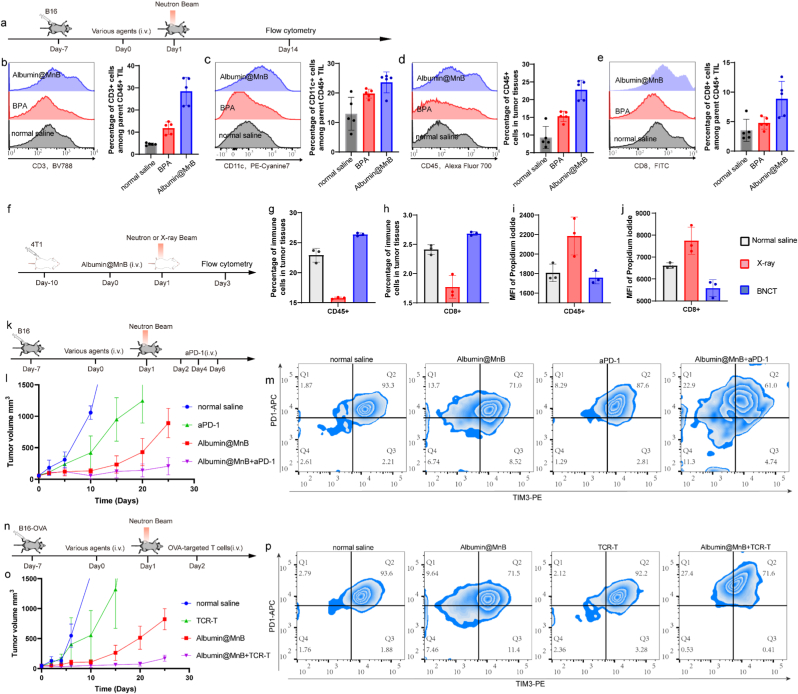
Synergistic antitumor efficacy of Albumin@MnB-based BNCT with T-Cell immunotherapy. a, Experimental timeline. Mice were inoculated with B16 cells 7 days before the start of the experiment. On Day 0, mice received narmal saline, BPA or Albumin@MnB treatments via intravenous injection (i.v.). On Day 1, mice were subjected to neutron beam irradiation, and were sacrificed on Day 14. Tissues were processed and analyzed by flow cytometry. **b-e**, Percentage of CD3^+^ cells among CD45^+^ tumor-infiltrating leukocytes (TILs) (b). Percentage of CD11c + cells among CD45^+^ TILs (c). Flow cytometry analysis of the percentage of CD45^+^ cells in tumor tissues (d). Percentage of CD8^+^ cells among CD45^+^ TILs (e). **f**, Experimental timeline. Mice were inoculated with 4T1 cells 10 days before the start of the experiment. On Day 0, mice received Albumin@MnB treatments via intravenous injectioni (i.v.). On Day 1, mice were subjected to neutron or X-ray beam irradiation, and were sacrificed on Day 14. Tissues were processed and analyzed by flow cytometry. **g-j**, Percentage of CD45 in immune cells from tumor tissues (g) and mean fluorescence intensity (MFI) of PI staining (h), as well as the percentage of CD8 in immune cells from tumor tissues (i) and MFI of PI staining (j). **k**, Experimental timeline. Mice were inoculated with B16 cells 7 days before the start of the experiment. On Day 0, mice received aPD-1, Albumin@MnB or aPD-1 + Albumin@MnB treatments via intravenous injection (i.v.). On Day 1, mice were subjected to neutron beam irradiation. Starting from Day 2, anti-PD-1 antibody treatment was administered via intravenous injection every other day (on Days 4 and 6). **l**, Tumor growth curves in mice. **m**, Flow cytometry analysis of the proportion of exhausted T cells (PD-1+TIM-3+) in tumors. **n**, Experimental timeline. Mice were inoculated with B16-OVA cells 7 days before the start of the experiment. On Day 0, mice received TCR-T, Albumin@MnB or TCR-T + Albumin@MnB treatments via intravenous injectioni (i.v.). On Day 1, mice were subjected to neutron beam irradiation. On Day 2, mice received OVA-targeted T cells via intravenous injection. **o**, Tumor growth curves in mice. **p**, Flow cytometry analysis of the proportion of exhausted T cells (PD- 3761+TIM-3+) in tumors.

Building upon the therapeutic synergy between Albumin@MnB and neutron irradiation, we conducted comprehensive profiling of post-BNCT tumor immune microenvironments to elucidate key immunological mechanisms (Fig. 10a-e). Quantitative analysis revealed enhanced infiltration of CD45^+^ leukocytes across all treatment groups (BPA: 18.4% vs saline: 10.1%; Albumin@MnB: 28.7%, Fig. 10d). Notably, Albumin@MnB-treated tumors exhibited significantly elevated proportions of CD3^+^ T cells (Fig. 10b), CD11c + dendritic cells (Fig. 10c), and CD8^+^ T cells (Fig. 10e) compared to BPA-treated counterparts.

Comparative analysis revealed Albumin@MnB-BNCT's superiority over conventional X-ray radiotherapy in both tumor control (Fig. 10f, S6-7) and immune preservation (Fig. 10g -j). Cellular-scale precision of BNCT better maintained tumor-associated immune populations versus X-irradiation (Fig. 10 g, h), as quantified by reduced propidium iodide staining (Fig. 10 i, j).

Given the clinical success of T cell-centric immunotherapies (e.g., immune checkpoint inhibitors, adoptive TCR-T therapies), we engineered a combinatorial strategy integrating Albumin@MnB-mediated BNCT with immunomodulation. Previous systematic summaries of NCT have confirmed the feasibility of combining targeted radiotherapy with immune regulation, laying a foundation for the development of multimodal therapeutic strategies [52]. While PD-1 blockade or Albumin@MnB-BNCT monotherapy showed moderate B16 melanoma control (Fig. 10k-m), their combination achieved synergistic tumor control with sustained suppression and minimal recurrence. Flow cytometric analysis demonstrated Albumin@MnB monotherapy substantially reduced exhausted T cell populations (PD-1+TIM-3+), an effect potentiated by αPD-1 coadministration (Fig. 10m). In OVA-expressing B16 models with adoptive TCR-T transfer (Fig. 10n-p), Albumin@MnB-BNCT synergized with cellular immunotherapy to mitigate T cell exhaustion (Fig. 10p), maintaining exhaustion levels comparable to monotherapy. This preservation of T cell functionality suggests Albumin@MnB may prime innate immune activation to reshape the immunosuppressive TME.

These findings position Albumin@MnB-BNCT as a multimodal platform bridging precision radiobiology with adaptive immunity, addressing two fundamental barriers in cancer therapy: treatment resistance and immune evasion through its dual-pronged mechanism of direct tumor eradication and immunogenic modulation.

## 4 Conclusion

This study establishes boron neutron capture immunotherapy (BNCI) as a transformative paradigm in precision radio-immunotherapy through the rational design of Albumin@MnB—a tumor-targeted nanoplatform integrating neutron capture, immunomodulation, and theranostic capabilities. By leveraging ultra-low-cost borax-derived^10^B and radiation-responsive Mn^2+^, we overcome two fundamental limitations of conventional BNCT: achieving tumor regression at lower clinical boron dose, and converting neutron-induced cell damage into systemic antitumor immunity. The platform's intrinsic MRI contrast enables closed-loop treatment optimization through real-time pharmacokinetic monitoring and neutron dose modulation, resolving longstanding challenges in BNCT precision delivery. Crucially, Albumin@MnB creates *in situ* vaccination effects that potentiate adoptive T-cell therapies and αPD-L1 checkpoint blockade, achieving metastatic tumor eradication in combinatorial regimens. This work redefines BNCT from a localized radiolytic modality to a multifunctional immunotherapeutic platform, where tumor-specific radiation damage and immunogenic cell death are synergistically weaponized. The BNCI strategy bridges three critical domains in oncology: precision radiobiology, immune microenvironment engineering, and image-guided personalized medicine, offering a clinically translatable blueprint for next-generation radio-immunotherapies.

## CRediT authorship contribution statement

**Chang Chen:** Data curation, Formal analysis, Writing – original draft. **Chao Wang:** Formal analysis, Software, Writing – original draft. **Lin Zhang:** Data curation, Formal analysis, Writing – original draft. **Yue Yu:** Data curation, Formal analysis. **Xu Li:** Methodology, Software. **Wenhao Pan:** Investigation. **Yuanyu Liu:** Methodology, Software. **Jiheng Wang:** Investigation. **Maosong Yang:** Investigation. **Bing Hong:** Methodology. **Xingguang Hu:** Visualization. **Jichao Wang:** Visualization. **Yuzhong Qian:** Methodology, Validation. **Xiancai Meng:** Methodology, Validation. **Yinghuai Zhu:** Writing – review & editing. **Zhigang Liu:** Writing – review & editing. **Shaobo Huang:** Methodology, Validation. **Lizheng Liang:** Resources, Supervision, Writing – review & editing. **Jinhui Wu:** Resources, Validation, Writing – review & editing. **Yang Liu:** Conceptualization, Project administration, Supervision, Visualization, Writing – review & editing.

## Declaration of Competing Interest

The authors declare that they have no known competing financial interests or personal relationships that could have appeared to influence the work reported in this paper.

## Data Availability

Data will be made available on request.
