# Supplementary material for: Systemic Immunity Triggered by Boron Neutron Capture Therapy via Manganese-Borate Complex
Source: Mater Today Bio. 2026 Feb 5;37:102904. doi: 10.1016/j.mtbio.2026.102904 (PMC12930067; doi:10.1016/j.mtbio.2026.102904)
Supplement: Multimedia component 1 [file mmc1.docx]

Supporting Information

1. **Supplementary Figure**


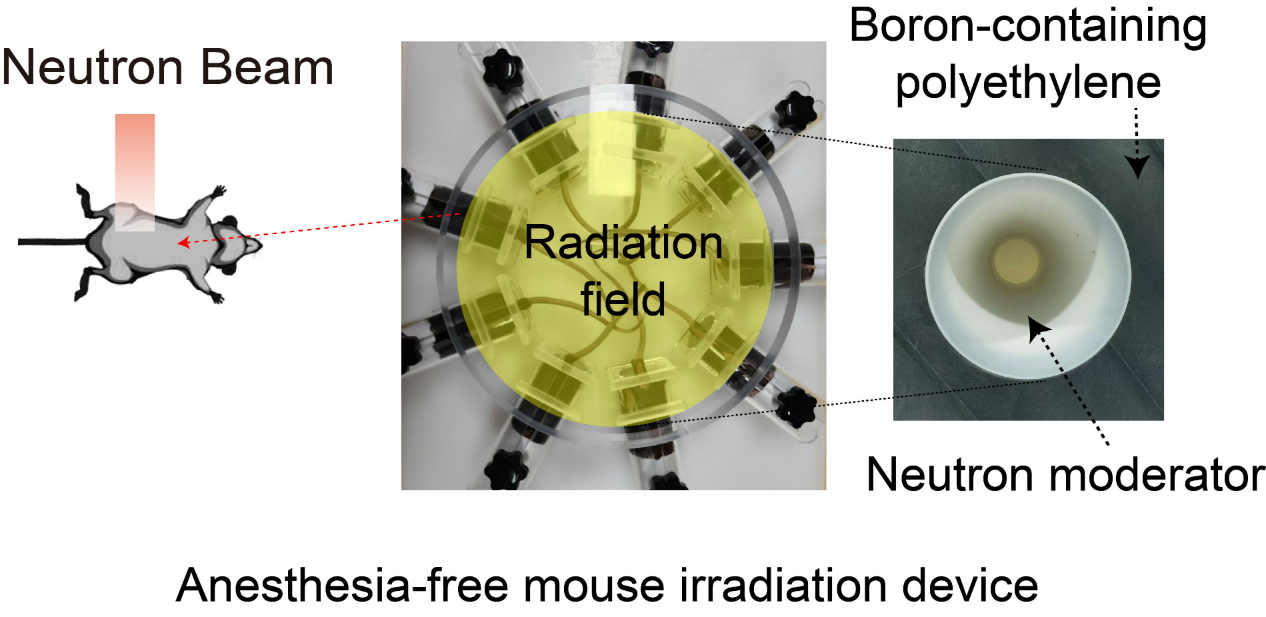


**Figure S1.** Anesthesia-free mouse irradiation device

**
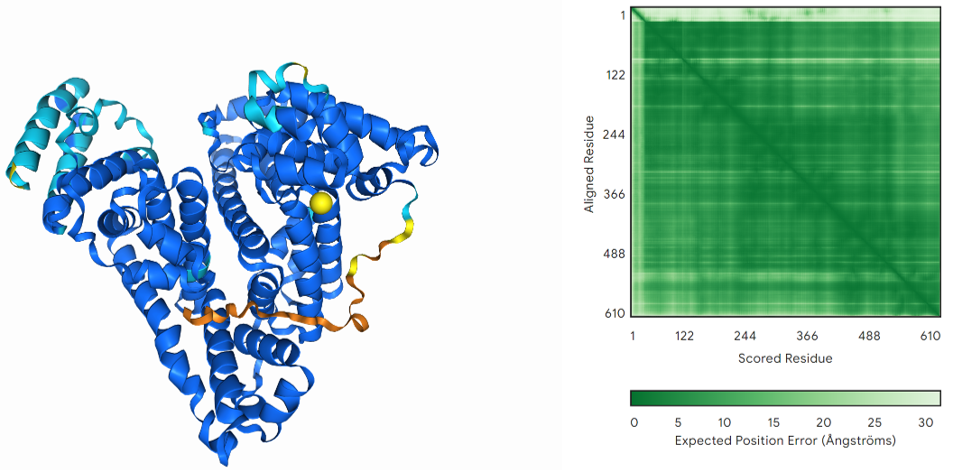
**

**Figure S2.** Predicted Binding of Manganese Ions to Albumin


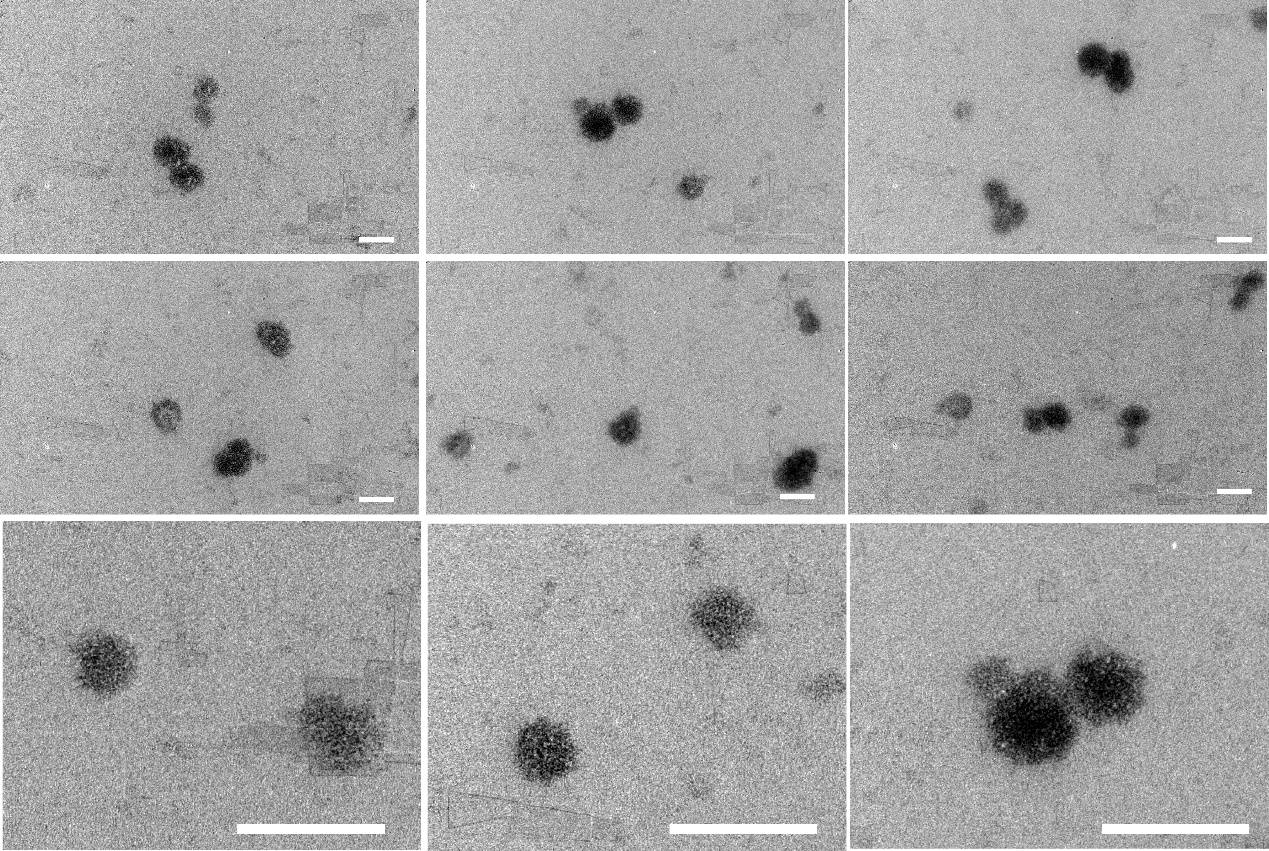


**Figure S3.** The TEM image of Alb@MnB protein nanoparticles. (Scale bars, 200 nm)


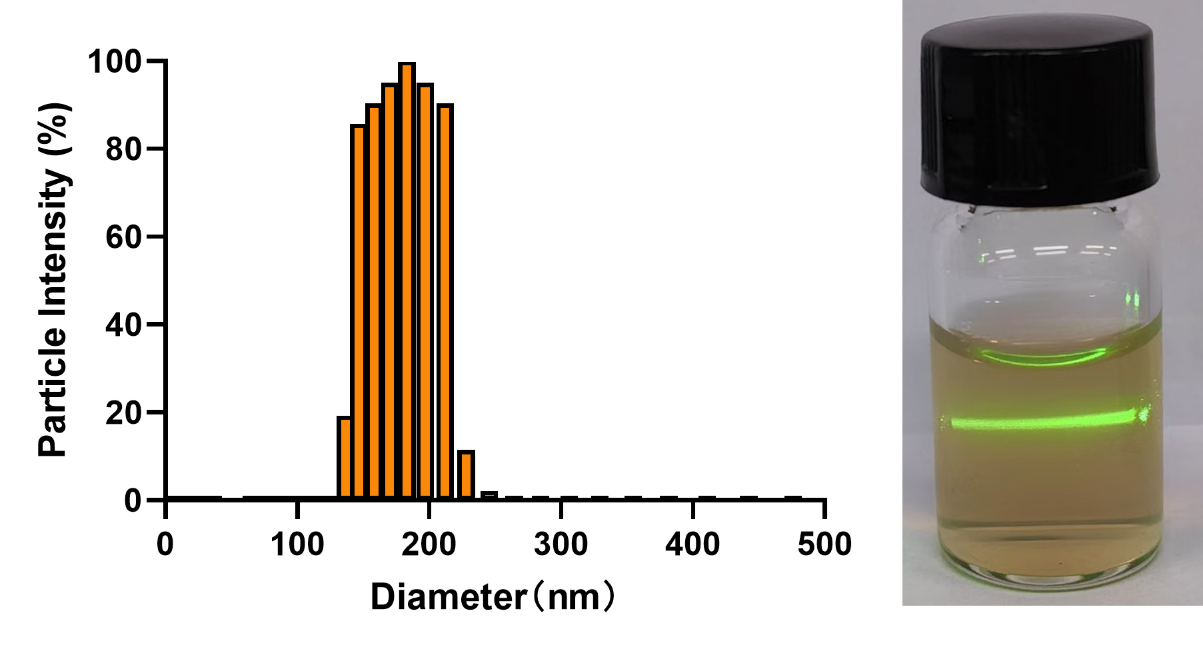


**Figure S4.** Visual representation and particle size analysis of Alb@MnB.


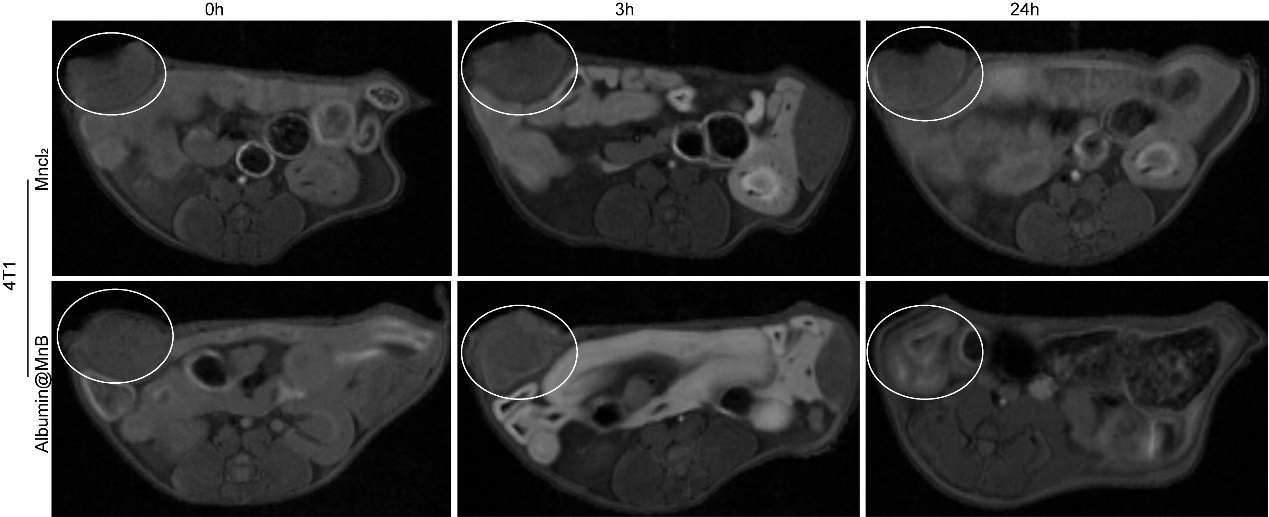


**Figure S5.** 9.4T MRI imaging showing the distribution of MnCl_2_ and Albumin@MnB in the same mice at 0h, 3h, and 24h post-injection.


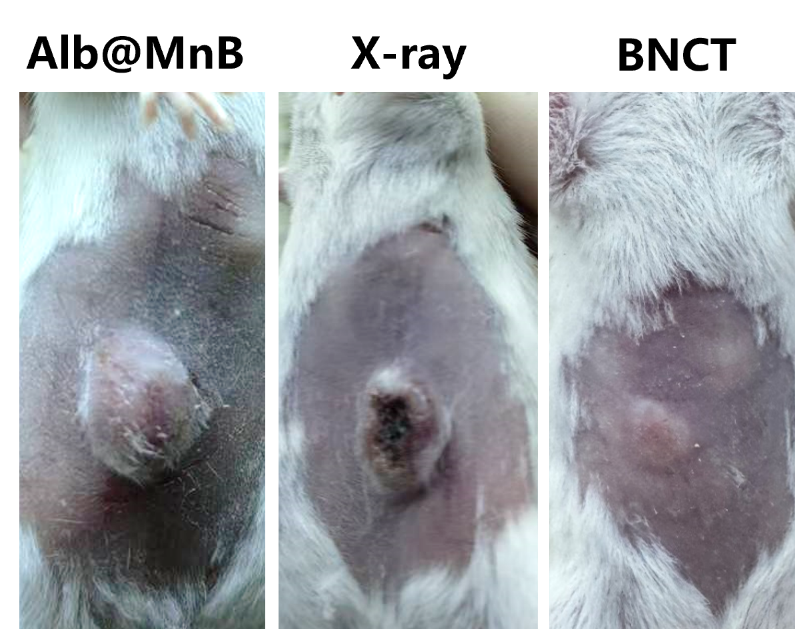


**Figure S6.** Photographs of tumors in model mice treated with Albumin@MnB, X-ray, or BNCT.


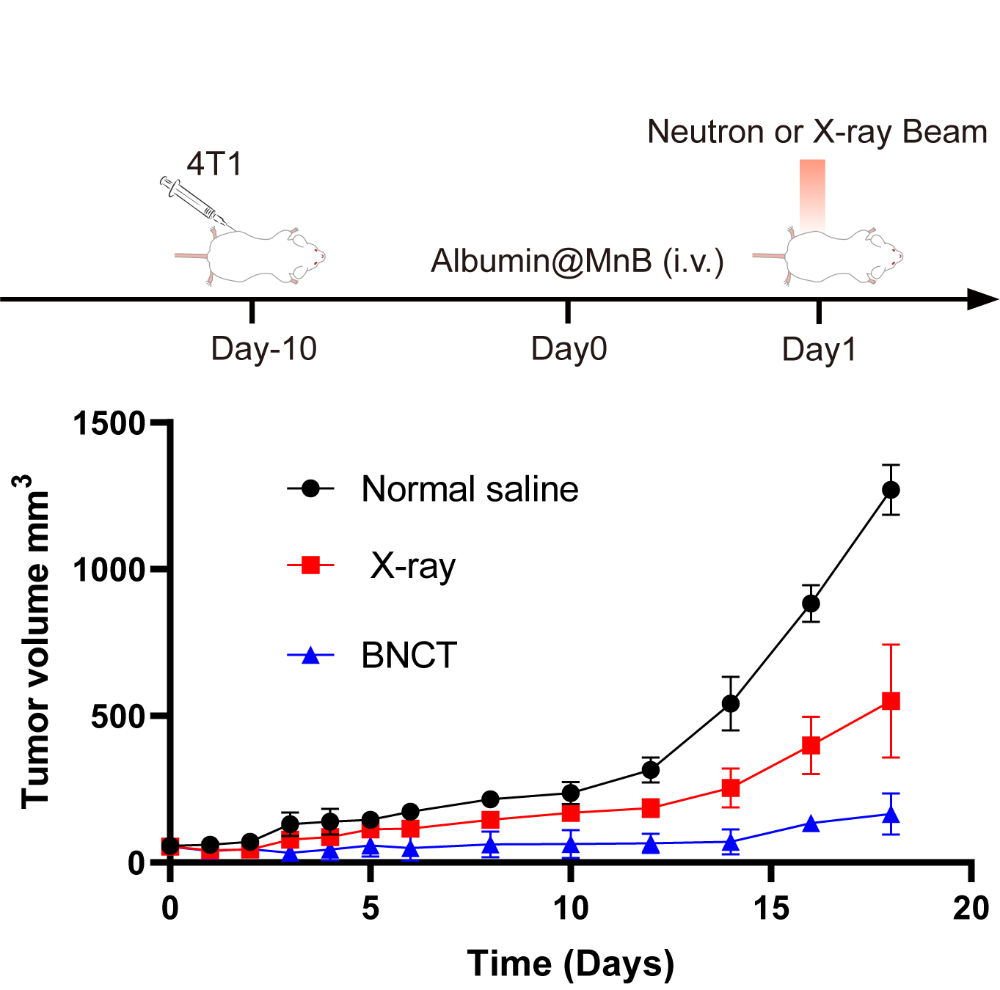


**Figure S7.** Experimental timeline: Mice were inoculated with 4T1 cells 10 days before the start of the experiment. On Day 0, mice received Albumin@MnB treatments via intravenous injectioni (i.v.). On Day 1, mice were subjected to neutron or X-ray beam irradiation, and were sacrificed on Day 14. Tumor growth curves in mice.


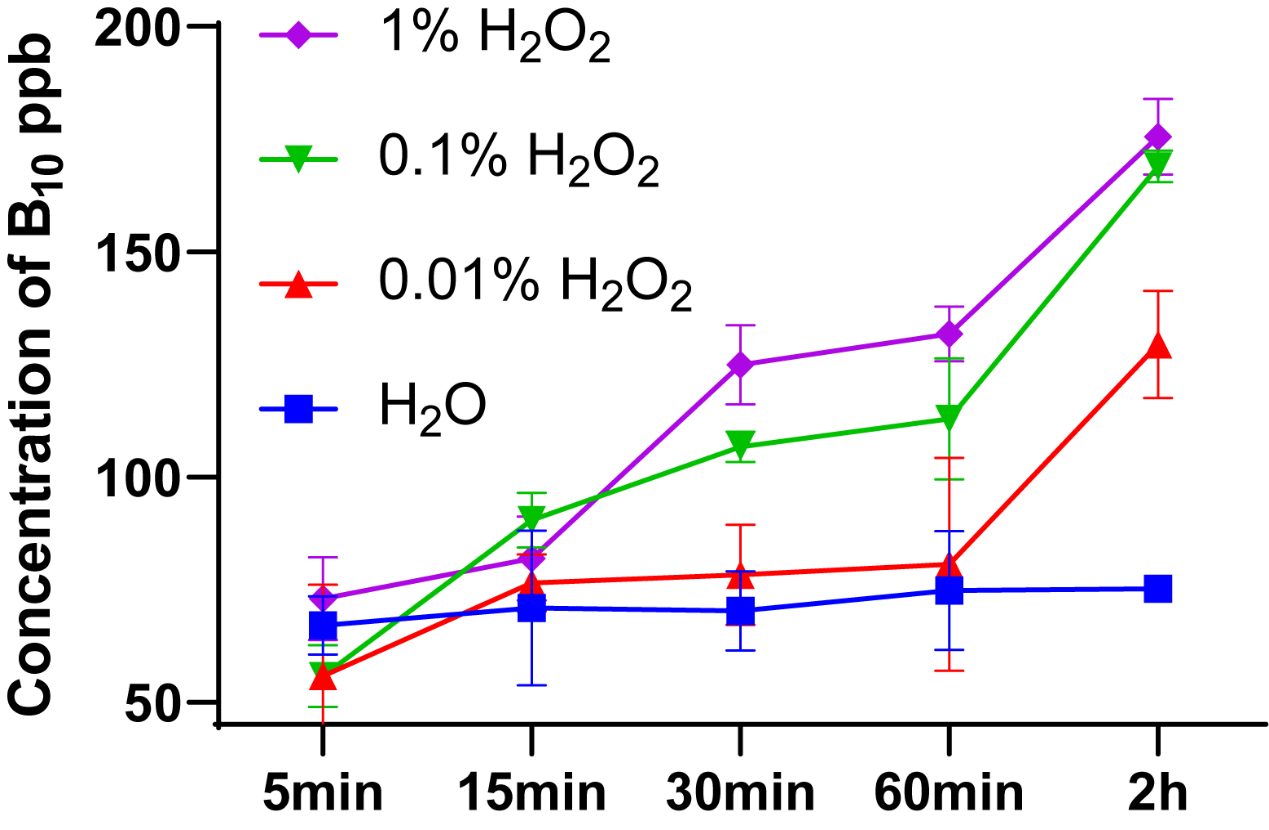


**Figure S8.** Release of boron from the protein formulation at different H₂O₂ concentrations (n=3, Data represent mean ± s.d.)


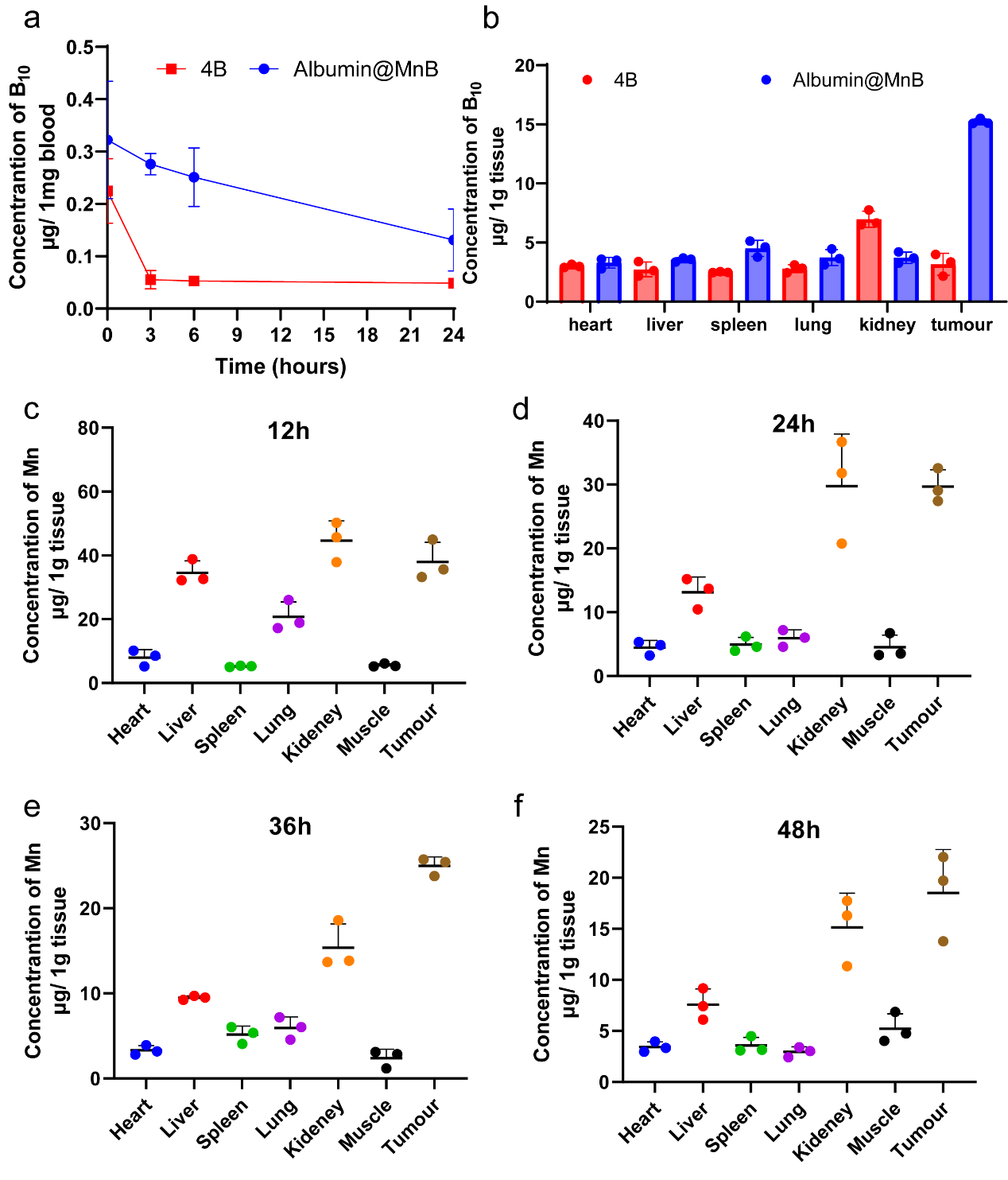


**Figure S9. Biodistribution of Boron and Manganese.** a, Blood circulation profiles of Albumin@MnB and 4B. b, Tissue distribution profiles of Albumin@MnB and 4B. c–f, Manganese (Mn) concentrations (μg/g tissue) in major organs (heart, liver, spleen, lung, kidney, and muscle) and tumor tissues of mice at 12 h (c), 24 h (d), 36 h (e), and 48 h post tail vein injection of Albumin@MnB. (n=3, Data represent mean ± s.d.)


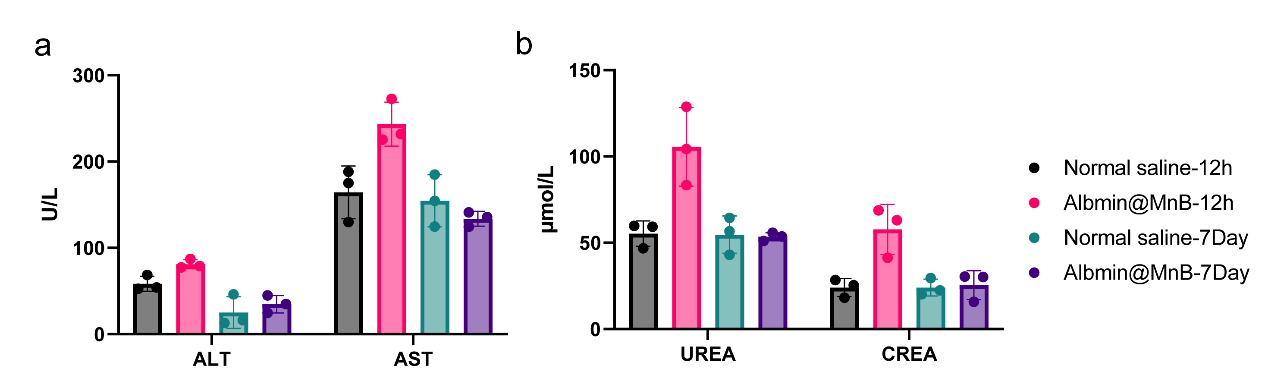


**Figure S10. Biological Safety Evaluation of Albumin@MnB.** a, Assays of liver function-related serum biomarkers (ALT and AST) in mice at different time points post-injection. b, Levels of renal function-related serum biomarkers, namely blood urea nitrogen (BUN) and creatinine, in mice at different time points post-injection.

**2. Supplementary Table**

Table S1 Sequence List of PCR Primers

| Oligo Name | Sequence |
| --- | --- |
| IFNb-F | TATAAGCAGCTCCAGCTCC |
| IFNb-R | ACAACAATAGTCTCATTCCACC |
| CXCL10-F | CGTCATTTTCTGCCTCATCC |
| CXCL10-R | CAGACATCTCTGCTCATCATTC |
| GAPDH-F | ACAGTCCATGCCATCACTGC |
| GAPDH-R | TTCAGCTCTGGGATGACCTT |

**Table S2** The differential metabolites in 4T1 cells before and after Nano intervention.

| **No** | **Metabolite** | **Formula** | ***m*/*z*** | **Rt (s)** | ***P*-Value** | **Q-Value** | **Fold Change** | **VIP** |
| --- | --- | --- | --- | --- | --- | --- | --- | --- |
| 1 | cis-Aconitic acid | C_6_H_6_O_6_ | 173.0099 | 251 | 0.04 | 0.57 | 1.60 | 1.69 |
| 2 | N-Acetyl-beta-alanine | C_5_H_9_NO_3_ | 130.0515 | 169 | 0.02 | 0.44 | 1.54 | 1.74 |
| 3 | Uridine | C_9_H_12_N_2_O_6_ | 243.0631 | 68.2 | 0.04 | 0.56 | 1.24 | 1.69 |
| 4 | Creatine | C_4_H_9_N_3_O_2_ | 132.0778 | 206.8 | 0.02 | 0.47 | 1.37 | 1.80 |
| 5 | Cytidine 5'-monophosphate (CMP) | C_9_H_14_N_3_O_8_P | 324.0622 | 264.7 | 0.04 | 0.57 | 1.93 | 1.96 |
| 6 | Uridine 5'-monophosphate (UMP) | C_9_H_13_N_2_O_9_P | 323.0303 | 255.5 | 0.04 | 0.57 | 2.62 | 1.86 |
| 7 | N-Acetylalanine | C_5_H_9_NO_3_ | 130.0514 | 151.2 | 0.03 | 0.50 | 1.32 | 1.73 |
| 8 | beta-Guanidinopropionic acid | C_4_H_9_N_3_O_2_ | 132.0778 | 206.8 | 0.02 | 0.47 | 1.37 | 1.80 |
| 9 | Cytidine 5'-diphosphocholine (CDP-choline) | C_14_H_26_N_4_O_11_P_2_ | 489.1196 | 254.8 | 0.00 | 0.11 | 1.65 | 2.00 |
| 10 | 3-Hydroxybutyrylcarnitine (Car(4:0-O)) | C_11_H_21_NO_5_ | 248.1515 | 192.3 | 0.02 | 0.43 | 1.66 | 1.86 |
| 11 | Acetylcarnitine (Car(2:0)) | C_9_H_17_NO_4_ | 204.1248 | 183.7 | 0.00 | 0.18 | 1.84 | 1.99 |
| 12 | Propionylcarnitine (Car(3:0)) | C_10_H_19_NO_4_ | 218.1407 | 170.4 | 0.01 | 0.35 | 1.51 | 1.84 |
| 13 | PC(16:0/20:4) | C_44_H_80_NO_8_P | 762.5374 | 65.6 | 0.05 | 0.61 | 1.23 | 1.68 |
| 14 | Car(18:0) | C_25_H_49_NO_4_ | 428.3779 | 93.7 | 0.04 | 0.58 | 1.31 | 1.69 |
| 15 | 4-Hydroxyproline | C_5_H_9_NO_3_ | 130.0514 | 151.2 | 0.03 | 0.50 | 1.32 | 1.73 |
| 16 | 5-Aminolevulinic acid | C_5_H_9_NO_3_ | 130.0514 | 151.2 | 0.03 | 0.50 | 1.32 | 1.73 |
| 17 | 3-Methoxy[1]benzothieno[2,3-c]quinolin-6(5H)-one | C_16_H_11_NO_2_S | 280.0435 | 69.5 | 0.02 | 0.47 | 1.21 | 1.80 |
| 18 | Artocarpin | C_26_H_28_O_6_ | 437.1977 | 17.8 | 0.04 | 0.58 | 1.74 | 1.72 |
| 19 | 1,2-Dilinoleoyl-sn-glycero-3-phosphoethanolamine | C_41_H_74_NO_8_P | 740.5276 | 38.5 | 0.05 | 0.60 | 1.16 | 1.69 |
| 20 | (1Z)-1,2-Naphthalenedione 1-(3-pyridinylhydrazone) | C_15_H_11_N_3_O | 250.0958 | 273.7 | 0.00 | 0.12 | 1.63 | 1.99 |
| 21 | 4-(1,3-Dioxo-1,3-dihydro-2H-isoindol-2-yl)-2-hydroxybenzoic acid | C_15_H_9_NO_5_ | 282.0404 | 69.5 | 0.02 | 0.44 | 1.21 | 1.83 |
| 22 | Pro-Glu | C_10_H_16_N_2_O_5_ | 245.1155 | 240.8 | 0.02 | 0.43 | 1.51 | 1.83 |
| 23 | 1-Hexadecyl-2-(9Z-octadecenoyl)-sn-glycero-3-phosphoethanolamine | C_39_H_78_NO_7_P | 702.5438 | 37.9 | 0.01 | 0.39 | 3.93 | 1.16 |
| 24 | Dynasore | C_18_H_14_N_2_O_4_ | 321.089 | 244.4 | 0.04 | 0.58 | 1.17 | 1.71 |
| 25 | PC(20:1(11Z)/P-16:0) | C_44_H_86_NO_7_P | 772.627 | 108.7 | 0.02 | 0.45 | 1.76 | 1.68 |
| 26 | N-(3-Dimethylaminopropyl)-N'-ethylcarbodiimide | C_8_H_17_N_3_ | 156.1509 | 198.5 | 0.04 | 0.56 | 4.07 | 1.49 |
| 27 | Uracil | C_4_H_4_N_2_O_2_ | 111.0203 | 34.6 | 2.05 | 9.78 ×10^− 6^ | 0.03 | 0.52 |
| 28 | Pyroglutamic acid | C_5_H_7_NO_3_ | 128.0358 | 178.9 | 1.74 | 0.03 | 0.50 | 0.66 |
| 29 | Thymine | C_5_H_6_N_2_O_2_ | 125.0361 | 30.8 | 2.04 | 1.10×10^− 5^ | 0.03 | 0.27 |
| 30 | Pyridoxal (Vitamin B6) | C_8_H_9_NO_3_ | 166.0514 | 38.2 | 1.78 | 0.03 | 0.54 | 0.66 |
| 31 | Docebenone | C_21_H_26_O_3_ | 325.1806 | 23.4 | 1.75 | 0.02 | 0.47 | 0.62 |
| 32 | Pyridoxine | C_8_H_11_NO_3_ | 168.0671 | 40.2 | 1.98 | 1.34×10^− 4^ | 0.15 | 0.53 |
| 33 | cis-11-Eicosenoic acid | C_20_H_38_O_2_ | 309.281 | 19.3 | 1.85 | 0.01 | 0.36 | 0.79 |
| 34 | 11-Dehydrocorticosterone | C_21_H_28_O_4_ | 325.1806 | 23.4 | 1.75 | 0.02 | 0.47 | 0.62 |
| 35 | sn-Glycerol 3-phosphate | C_3_H_9_O_6_P | 171.0072 | 252.6 | 1.82 | 0.02 | 0.46 | 0.45 |
| 36 | Pseudouridine | C_9_H_12_N_2_O_6_ | 243.0633 | 139 | 1.92 | 0.01 | 0.36 | 0.61 |
| 37 | Guanine | C_5_H_5_N_5_O | 150.0426 | 133.1 | 1.75 | 0.02 | 0.44 | 0.66 |
| 38 | Ribothymidine | C_10_H_14_N_2_O_6_ | 257.0789 | 54.4 | 2.03 | 3.45×10^− 4^ | 0.09 | 0.44 |
| 39 | PC(P-16:0/0:0) | C_24_H_50_NO_6_P | 480.349 | 112.4 | 2.04 | 5.72×10^− 5^ | 0.06 | 0.71 |
| 40 | Acadesine (Drug) | C_9_H_14_N_4_O_5_ | 259.1059 | 104.9 | 1.83 | 0.02 | 0.46 | 0.52 |
| 41 | Cytosine | C_4_H_5_N_3_O | 112.0515 | 102.7 | 1.92 | 0.01 | 0.33 | 0.50 |
| 42 | Ectoine | C_6_H_10_N_2_O_2_ | 143.0828 | 198.4 | 1.92 | 0.01 | 0.35 | 0.71 |
| 43 | LPC(16:0) | C_24_H_50_NO_7_P | 496.344 | 122.2 | 2.04 | 7.99×10^− 5^ | 0.06 | 0.44 |
| 44 | alpha-CEHC (alpha-Carboxyethyl hydrochroman) | C_16_H_22_O_4_ | 277.1453 | 24.2 | 1.69 | 0.04 | 0.58 | 0.76 |
| 45 | 7-Methylguanine | C_6_H_7_N_5_O | 166.0737 | 105 | 1.97 | 2.91×10^− 3^ | 0.21 | 0.44 |
| 46 | N-Acetylglucosamine 6-Phosphate | C_8_H_16_NO_9_P | 300.0505 | 260.2 | 2.03 | 1.47×10^− 4^ | 0.07 | 0.64 |
| 47 | 1-O-Hexadecyl-sn-glycero-3-phosphocholine (LPC(O-16:0/0:0)) | C_24_H_52_NO_6_P | 482.3647 | 125.9 | 2.03 | 8.79×10^− 5^ | 0.06 | 0.50 |
| 48 | 1-Myristoyl-sn-glycero-3-phosphocholine (LPC(14:0/0:0)) | C22H46NO7P | 468.3128 | 125.4 | 2.04 | 3.09×10^− 4^ | 0.09 | 0.45 |
| 49 | LPC(18:2/0:0) | C_26_H_50_NO_7_P | 520.3447 | 115.5 | 1.81 | 0.03 | 0.50 | 0.49 |
| 50 | 3-Methyluridine | C_10_H_14_N_2_O_6_ | 257.0789 | 54.4 | 2.03 | 3.45×10^− 4^ | 0.09 | 0.44 |
| 51 | Daminozide | C_6_H_12_N_2_O_3_ | 143.0828 | 198.4 | 1.92 | 0.01 | 0.35 | 0.71 |
| 52 | 1-Methylpseudouridine | C_10_H_14_N_2_O_6_ | 257.0789 | 54.4 | 2.03 | 3.45×10^− 4^ | 0.09 | 0.44 |
| 53 | 2-Hydroxyadenine | C_5_H_5_N_5_O | 150.0426 | 133.1 | 1.75 | 0.02 | 0.44 | 0.66 |
| 54 | LPC(18:0/0:0) | C_26_H_54_NO_7_P | 524.3755 | 119.4 | 2.04 | 6.14×10^− 5^ | 0.06 | 0.53 |
| 55 | N-Acetylmuramic acid | C_11_H_19_NO_8_ | 276.1109 | 152.4 | 1.98 | 3.76×10^− 3^ | 0.24 | 0.55 |
| 56 | Flopropione | C_9_H_10_O_4_ | 181.0487 | 199.7 | 1.94 | 0.01 | 0.29 | 0.54 |
| 57 | Nivalenol | C_15_H_20_O_7_ | 335.1092 | 176.1 | 1.82 | 0.02 | 0.44 | 0.66 |
| 58 | FA 18:3+2O | C_18_H_30_O_4_ | 309.2057 | 22.9 | 1.78 | 0.02 | 0.42 | 0.63 |
| 59 | 9S,15S-Dihydroxy-5Z,13E-prostadienoic acid | C_20_H_34_O_4_ | 337.2373 | 22.5 | 1.89 | 0.01 | 0.41 | 0.72 |
| 60 | Cohumulone | C_20_H_28_O_5_ | 349.2008 | 286.8 | 1.73 | 0.03 | 0.54 | 0.70 |
| 61 | (Z)-6-Octadecenoic acid | C_18_H_34_O_2_ | 281.2495 | 19.4 | 1.94 | 3.11×10^− 3^ | 0.22 | 0.71 |
| 62 | cis-4,10,13,16-Docosatetraenoic acid | C_22_H_36_O_2_ | 331.2654 | 18.6 | 1.72 | 0.05 | 0.59 | 0.74 |
| 63 | Isobutyric_acid | C_4_H_8_O_2_ | 87.0454 | 50.5 | 1.98 | 2.95×10^− 3^ | 0.21 | 0.61 |
| 64 | 4-(4-Nitrophenyl)-1H-imidazole | C_9_H_7_N_3_O_2_ | 188.0459 | 125.7 | 1.88 | 0.02 | 0.47 | 0.58 |
| 65 | 2-(4-Morpholinyl)-4H-pyrimido[2,1-a]isoquinolin-4-one | C_16_H_15_N_3_O_2_ | 282.1223 | 170.9 | 1.87 | 0.01 | 0.34 | 0.59 |
| 66 | Zederone | C_15_H_18_O_3_ | 247.131 | 50.1 | 1.95 | 0.01 | 0.29 | 0.43 |
| 67 | 5-Amino-1-pentanol | C_5_H_13_NO | 104.1078 | 162.6 | 1.82 | 0.02 | 0.45 | 0.71 |
| 68 | 8-Hydroxyguanine | C_5_H_5_N_5_O_2_ | 168.053 | 133.3 | 1.64 | 0.04 | 0.56 | 0.57 |
| 69 | 1-O-Octadecyl-sn-glyceryl-3-phosphorylcholine | C_26_H_56_NO_6_P | 510.3961 | 122.9 | 2.03 | 1.14×10^− 4^ | 0.07 | 0.52 |
| 70 | LPC(O-18:1) | C_26_H_54_NO_6_P | 508.3804 | 123.7 | 1.91 | 4.64×10^− 3^ | 0.25 | 0.55 |
| 71 | LPC(18:1) | C_26_H_52_NO_7_P | 522.3599 | 119.5 | 2.04 | 1.14×10^− 4^ | 0.07 | 0.48 |
| 72 | LPE(P-16:0) | C_21_H_44_NO_6_P | 438.3018 | 117 | 1.85 | 0.01 | 0.38 | 0.78 |
| 73 | 1-(1Z-Octadecenyl)-sn-glycero-3-phosphocholine | C_26_H_54_NO_6_P | 508.3805 | 108.8 | 1.99 | 2.05×10^− 3^ | 0.19 | 0.78 |
| 74 | 1,3-Diphenyl-2-propanone | C_15_H_14_O | 211.1096 | 49.9 | 1.83 | 0.01 | 0.32 | 0.46 |
| 75 | 17-Acetoxygrindelic acid | C_22_H_34_O_5_ | 377.2379 | 22.5 | 1.87 | 0.01 | 0.33 | 0.69 |
| 76 | 20-hydroxy_LTB4 | C_20_H_32_O_5_ | 351.2221 | 22.8 | 1.85 | 0.01 | 0.41 | 0.62 |
| 77 | Hydroxyprolyl-Proline | C_10_H_16_N_2_O_4_ | 229.1203 | 50 | 1.94 | 0.01 | 0.32 | 0.41 |
| 78 | Pyrovalerone | C_16_H_23_NO | 246.1835 | 312.6 | 1.84 | 0.01 | 0.40 | 0.76 |
| 79 | Isopropalin | C_15_H_23_N_3_O_4_ | 310.1792 | 248.6 | 1.80 | 0.02 | 0.45 | 0.64 |
| 80 | Thioridazine | C_21_H_26_N_2_S_2_ | 371.1665 | 14.6 | 1.68 | 0.05 | 0.59 | 0.79 |
| 81 | 4,6-Dichloro-1H-indole-2-carboxylic acid | C_9_H_5_Cl_2_NO_2_ | 227.9615 | 179 | 1.28 | 0.04 | 0.56 | 0.34 |
| 82 | [3-(Trifluoromethyl)-1H-pyrazol-1-yl]acetic acid | C_6_H_5_F_3_N_2_O_2_ | 193.0236 | 34.2 | 1.78 | 0.02 | 0.43 | 0.61 |
| 83 | LysoPC(18:3(6Z,9Z,12Z)) | C_26_H_48_NO_7_P | 518.3263 | 121.5 | 2.02 | 4.91×10^− 4^ | 0.10 | 0.49 |
| 84 | PC(P-18:1(9Z)/0:0) | C_26_H_52_NO_6_P | 506.3649 | 110.6 | 2.04 | 4.11×10^− 5^ | 0.05 | 0.62 |
| 85 | 1-Trifluoromethoxyphenyl-3-(1-propionylpiperidin-4-yl)urea | C_16_H_20_F_3_N_3_O_3_ | 360.1537 | 230.6 | 1.84 | 0.02 | 0.43 | 0.74 |
| 86 | 13-(Dodecan-2-yl)-6-(1-hydroxyethyl)-3-(hydroxymethyl)-12-methyl-9-(propan-2-yl)-1-oxa-4,7,10-triazacyclotridecane-2,5,8,11-tetrone | C_28_H_51_N_3_O_7_ | 540.369 | 125.7 | 1.99 | 1.47×10^− 3^ | 0.16 | 0.61 |
| 87 | Pyrrocidine B | C_31_H_39_NO_4_ | 490.2951 | 125.5 | 2.03 | 5.41×10^− 5^ | 0.06 | 0.42 |
| 88 | LysoPC(22:1(13Z)) | C_30_H_60_NO_7_P | 578.4232 | 114.4 | 1.99 | 1.21×10^− 3^ | 0.15 | 0.67 |
| 89 | Xerocomic_acid | C_18_H_12_O_8_ | 357.0592 | 250.7 | 1.83 | 0.02 | 0.45 | 0.77 |
| 90 | PC(16:1(9Z)/0:0)[U] | C_24_H_48_NO_7_P | 494.3286 | 122.4 | 1.96 | 1.37×10^− 3^ | 0.16 | 0.49 |
| 91 | Cedrin | C_16_H_14_O_8_ | 335.077 | 250.7 | 1.90 | 0.01 | 0.35 | 0.75 |
| 92 | Nitrofurazone | C_6_H_6_N_4_O_4_ | 197.0323 | 53.9 | 1.94 | 0.01 | 0.28 | 0.59 |
| 93 | Stigmastane-3,6-dione | C_29_H_48_O_2_ | 429.3766 | 16.7 | 1.68 | 0.03 | 0.54 | 0.63 |
| 94 | 1,7-Dioxa-4,10-diazacyclododecane | C_8_H_18_N_2_O_2_ | 175.1455 | 305.4 | 1.77 | 0.02 | 0.47 | 0.78 |
| 96 | Caryophyllene_epoxide | C_15_H_24_O | 221.1877 | 162.2 | 1.76 | 0.04 | 0.56 | 0.71 |
| 96 | LysoPE(P-18:1(9Z)/0:0) | C_23_H_46_NO_6_P | 462.3008 | 114.7 | 1.88 | 0.01 | 0.35 | 0.83 |
| 97 | M361T237 | C_13_H_24_N_6_O_6_ | 361.1867 | 236.6 | 1.90 | 0.01 | 0.35 | 0.52 |

**Table 3** The differential metabolites in 4T1 cells before and after BPA intervention.

| **No** | **Metabolite** | **Formula** | ***m*/*z*** | **Rt (s)** | ***P*-Value** | **Q-Value** | **Fold Change** | **VIP** |
| --- | --- | --- | --- | --- | --- | --- | --- | --- |
|  | Uracil | C_4_H_4_N_2_O_2_ | 111.0203 | 34.6 | 8.68×10^− 5^ | 0.02 | 0.60 | 1.84 |
|  | Pyroglutamic acid | C_5_H_7_NO_3_ | 128.0358 | 178.9 | 0.01 | 0.10 | 0.62 | 1.74 |
|  | Taurine | C_2_H_7_NO_3_S | 124.0078 | 175.1 | 3.30×10^− 3^ | 0.08 | 0.54 | 1.77 |
|  | Lactate | C_3_H_6_O_3_ | 89.0247 | 125.2 | 4.10×10^− 4^ | 0.04 | 0.26 | 1.84 |
|  | Thymine | C_5_H_6_N_2_O_2_ | 125.0361 | 30.8 | 8.53×10^− 6^ | 0.01 | 0.37 | 1.85 |
|  | 4-Methylquinolin-2-ol | C_10_H_9_NO | 158.0616 | 17.7 | 0.01 | 0.13 | 0.54 | 1.73 |
|  | myo-Inositol | C_6_H_12_O_6_ | 179.0568 | 224.8 | 0.01 | 0.10 | 0.55 | 1.78 |
|  | 3-Amino-4-methylpentanoic acid | C_6_H_13_NO_2_ | 130.0878 | 163.5 | 0.01 | 0.10 | 0.40 | 1.78 |
|  | Val-Ile | C_11_H_22_N_2_O_3_ | 229.1565 | 113.2 | 0.01 | 0.17 | 0.52 | 1.71 |
|  | Ile-Leu | C_12_H_24_N_2_O_3_ | 243.1724 | 100.2 | 4.49×10^− 3^ | 0.09 | 0.56 | 1.75 |
|  | Leu-Leu | C_12_H_24_N_2_O_3_ | 243.1724 | 100.2 | 4.49×10^− 3^ | 0.09 | 0.56 | 1.75 |
|  | Ureidosuccinic acid | C_5_H_8_N_2_O_5_ | 175.0368 | 241.2 | 0.02 | 0.19 | 0.37 | 1.67 |
|  | Pyruvate | C_3_H_4_O_3_ | 87.0091 | 48.4 | 1.89×10^− 3^ | 0.07 | 0.65 | 1.82 |
|  | Pyridoxine | C_8_H_11_NO_3_ | 168.0671 | 40.2 | 5.55×10^− 5^ | 0.02 | 0.23 | 1.85 |
|  | cis-11.14-Eicosadienoic acid | C_20_H_36_O_2_ | 307.2653 | 19.3 | 0.02 | 0.18 | 1.10 | 1.66 |
|  | cis-11-Eicosenoic acid | C_20_H_38_O_2_ | 309.281 | 19.3 | 0.03 | 0.25 | 1.10 | 1.62 |
|  | Docosahexaenoic acid (DHA) | C_22_H_32_O_2_ | 327.234 | 19.3 | 0.02 | 0.19 | 1.20 | 1.64 |
|  | Leu-Val | C_11_H_22_N_2_O_3_ | 229.1565 | 113.2 | 0.01 | 0.17 | 0.52 | 1.71 |
|  | Leu-Ile | C_12_H_24_N_2_O_3_ | 243.1724 | 100.2 | 4.49×10^− 3^ | 0.09 | 0.56 | 1.75 |
|  | 2-Aminoadipic acid | C_6_H_11_NO_4_ | 160.0622 | 237.6 | 2.22×10^− 3^ | 0.07 | 0.70 | 1.78 |
|  | Glutamate | C_5_H_9_NO_4_ | 146.0465 | 231.7 | 6.67×10^− 4^ | 0.04 | 0.31 | 1.83 |
|  | Proline | C_5_H_9_NO_2_ | 114.0565 | 184.6 | 0.03 | 0.23 | 0.48 | 1.79 |
|  | Isoleucine | C_6_H_13_NO_2_ | 130.0878 | 163.5 | 0.01 | 0.10 | 0.40 | 1.78 |
|  | Glutathione disulfide | C_20_H_32_N_6_O_12_S_2_ | 611.1479 | 279.2 | 0.02 | 0.23 | 0.71 | 1.64 |
|  | alpha-Ketoglutaric acid (alpha-KG) | C_5_H_6_O_5_ | 145.0148 | 216.7 | 5.01×10^− 4^ | 0.04 | 0.38 | 1.84 |
|  | Valine | C_5_H_11_NO_2_ | 116.0721 | 180.4 | 0.04 | 0.30 | 0.44 | 1.77 |
|  | Adenine | C_5_H_5_N_5_ | 134.0477 | 66.1 | 0.04 | 0.31 | 0.60 | 1.61 |
|  | 2-Aminopurine | C_5_H_5_N_5_ | 134.0477 | 66.1 | 0.04 | 0.31 | 0.60 | 1.61 |
|  | Glucose 6-phosphate | C_6_H_13_O_9_P | 259.0238 | 263.5 | 0.04 | 0.28 | 0.60 | 1.63 |
|  | sn-Glycerol 3-phosphate | C_3_H_9_O_6_P | 171.0072 | 252.6 | 0.03 | 0.24 | 0.55 | 1.66 |
|  | Methionine | C_5_H_11_NO_2_S | 148.0443 | 172.4 | 4.85×10^− 3^ | 0.09 | 0.48 | 1.79 |
|  | Dihydroorotic acid | C_5_H_6_N_2_O_4_ | 157.0261 | 172.4 | 4.45×10^− 3^ | 0.09 | 0.36 | 1.76 |
|  | Thr-Leu | C_10_H_20_N_2_O_4_ | 231.1359 | 142.9 | 0.02 | 0.21 | 0.55 | 1.67 |
|  | Allantoin | C_4_H_6_N_4_O_3_ | 157.0373 | 91.9 | 2.17×10^− 3^ | 0.07 | 0.49 | 1.81 |
|  | 2-Ketocaproic acid | C_6_H_10_O_3_ | 129.0561 | 26.2 | 0.02 | 0.17 | 0.56 | 1.73 |
|  | 3-Methyl-2-oxovaleric acid | C_6_H_10_O_3_ | 129.0561 | 26.2 | 0.02 | 0.17 | 0.56 | 1.73 |
|  | Ketoleucine | C_6_H_10_O_3_ | 129.0561 | 26.2 | 0.02 | 0.17 | 0.56 | 1.73 |
|  | Mannose 1-phosphate | C_6_H_13_O_9_P | 259.0238 | 263.5 | 0.04 | 0.28 | 0.60 | 1.63 |
|  | Carnitine | C_7_H_15_NO_3_ | 162.1137 | 208.6 | 0.03 | 0.26 | 0.71 | 1.77 |
|  | Glycerophosphoethanolamine | C_5_H_14_NO_6_P | 214.0495 | 229.6 | 0.01 | 0.15 | 0.65 | 1.70 |
|  | O-Acetylserine | C_5_H_9_NO_4_ | 146.0465 | 176.9 | 0.04 | 0.29 | 0.34 | 1.79 |
|  | Mannitol | C_6_H_14_O_6_ | 181.0724 | 168.1 | 0.01 | 0.17 | 0.46 | 1.74 |
|  | Pseudouridine | C_9_H_12_N_2_O_6_ | 243.0633 | 139 | 0.04 | 0.29 | 0.74 | 1.59 |
|  | Fructose 6-phosphate | C_6_H_13_O_9_P | 259.0238 | 263.5 | 0.04 | 0.28 | 0.60 | 1.63 |
|  | Fructose 1-phosphate | C_6_H_13_O_9_P | 259.0238 | 263.5 | 0.04 | 0.28 | 0.60 | 1.63 |
|  | Creatine | C_4_H_9_N_3_O_2_ | 132.0778 | 206.8 | 0.01 | 0.16 | 0.72 | 1.69 |
|  | Leucine | C_6_H_13_NO_2_ | 132.103 | 160.5 | 4.95×10^− 4^ | 0.04 | 0.45 | 1.84 |
|  | 4-Aminobutyric acid (GABA) | C_4_H_9_NO_2_ | 104.0715 | 218.5 | 5.91×10^− 4^ | 0.04 | 0.56 | 1.82 |
|  | Trehalose | C_12_H_22_O_11_ | 341.1105 | 230.2 | 0.03 | 0.23 | 0.42 | 1.81 |
|  | Malonic acid | C_3_H_4_O_4_ | 103.0041 | 228 | 0.01 | 0.10 | 0.73 | 1.74 |
|  | Tyrosine | C_9_H_11_NO_3_ | 180.0673 | 182.6 | 0.01 | 0.13 | 0.34 | 1.77 |
|  | Ribothymidine | C_10_H_14_N_2_O_6_ | 257.0789 | 54.4 | 4.47×10^− 4^ | 0.04 | 0.43 | 1.82 |
|  | Isomaltose | C_12_H_22_O_11_ | 341.1105 | 230.2 | 0.03 | 0.23 | 0.42 | 1.81 |
|  | 3-Hydroxypyruvic acid | C_3_H_4_O_4_ | 103.0041 | 228 | 0.01 | 0.10 | 0.73 | 1.74 |
|  | L-​Leucyl-​L-​alanine | C_9_H_18_N_2_O_3_ | 203.1408 | 142.5 | 0.02 | 0.20 | 0.50 | 1.70 |
|  | 2-Hydroxyglutaric acid | C_5_H_8_O_5_ | 147.0304 | 230.6 | 0.05 | 0.32 | 0.41 | 1.76 |
|  | Phenylalanine | C_9_H_11_NO_2_ | 164.0723 | 158.3 | 0.04 | 0.31 | 0.44 | 1.77 |
|  | Hydroxyphenyllactic acid | C_9_H_10_O_4_ | 181.0513 | 97.4 | 0.01 | 0.10 | 0.51 | 1.78 |
|  | Phenylacetylglycine | C_10_H_11_NO_3_ | 192.0673 | 97.2 | 0.02 | 0.22 | 0.36 | 1.81 |
|  | 2-Methylhippuric acid | C_10_H_11_NO_3_ | 192.0673 | 97.2 | 0.02 | 0.22 | 0.36 | 1.81 |
|  | Acadesine (Drug) | C_9_H_14_N_4_O_5_ | 259.1059 | 104.9 | 0.00 | 0.08 | 0.20 | 1.81 |
|  | Cytosine | C_4_H_5_N_3_O | 112.0515 | 102.7 | 0.04 | 0.31 | 0.61 | 1.78 |
|  | Norleucine | C_6_H_13_NO_2_ | 132.103 | 160.5 | 4.95×10^− 4^ | 0.04 | 0.45 | 1.84 |
|  | Guanosine 5'-monophosphate (GMP) | C_10_H_14_N_5_O_8_P | 364.069 | 267.5 | 0.01 | 0.16 | 0.65 | 1.71 |
|  | Phe-Ala | C_12_H_16_N_2_O_3_ | 237.1253 | 122.9 | 0.05 | 0.32 | 0.62 | 1.62 |
|  | LPC(16:0) | C_24_H_50_NO_7_P | 496.344 | 122.2 | 1.67×10^− 3^ | 0.06 | 0.65 | 1.79 |
|  | Creatinine | C_4_H_7_N_3_O | 114.0671 | 76.1 | 4.25×10^− 4^ | 0.04 | 0.48 | 1.83 |
|  | Tryptophan | C_11_H_12_N_2_O_2_ | 203.0833 | 160 | 0.01 | 0.13 | 0.28 | 1.78 |
|  | alpha-CEHC (alpha-Carboxyethyl hydrochroman) | C_16_H_22_O_4_ | 277.1453 | 24.2 | 1.17×10^− 3^ | 0.05 | 0.47 | 1.82 |
|  | beta-Guanidinopropionic acid | C_4_H_9_N_3_O_2_ | 132.0778 | 206.8 | 0.01 | 0.16 | 0.72 | 1.69 |
|  | Aspartate | C_4_H_7_NO_4_ | 134.046 | 238.2 | 0.01 | 0.11 | 1.70 | 1.68 |
|  | 4-Guanidinobutyric acid | C_5_H_11_N_3_O_2_ | 146.0936 | 211.6 | 2.34×10^− 3^ | 0.07 | 0.75 | 1.80 |
|  | Thiamine | C_12_H_17_N_4_OS | 265.114 | 205.9 | 2.37×10^− 3^ | 0.07 | 0.64 | 1.79 |
|  | Maltose | C_12_H_22_O_11_ | 365.1089 | 231.1 | 0.01 | 0.17 | 0.22 | 1.83 |
|  | gamma-Glutamylglutamic acid | C_10_H_16_N_2_O_7_ | 277.1057 | 263 | 0.03 | 0.26 | 0.67 | 1.59 |
|  | Cytidine 5'-diphosphocholine (CDP-choline) | C_14_H_26_N_4_O_11_P_2_ | 489.1196 | 254.8 | 1.71×10^− 3^ | 0.06 | 0.55 | 1.81 |
|  | Arg-Ala | C_9_H_19_N_5_O_3_ | 246.1584 | 243 | 0.01 | 0.12 | 0.42 | 1.76 |
|  | Prolylalanine | C_8_H_14_N_2_O_3_ | 187.1094 | 199.9 | 1.09×10^− 3^ | 0.05 | 0.61 | 1.82 |
|  | 3-Hydroxybutyrylcarnitine (Car(4:0-O)) | C_11_H_21_NO_5_ | 248.1515 | 192.3 | 1.78×10^− 3^ | 0.06 | 0.45 | 1.83 |
|  | Butyrylcarnitine (Car(4:0)) | C_11_H_21_NO_4_ | 232.1564 | 158.7 | 0.01 | 0.12 | 0.54 | 1.78 |
|  | N-Acetylglucosamine 6-Phosphate | C_8_H_16_NO_9_P | 300.0505 | 260.2 | 2.76×10^− 4^ | 0.04 | 0.81 | 1.84 |
|  | Isobutyrylcarnitine (Car(4:0)) | C_11_H_21_NO_4_ | 232.1564 | 158.7 | 0.01 | 0.12 | 0.54 | 1.78 |
|  | Glutamine | C_5_H_10_N_2_O_3_ | 147.0777 | 223.6 | 8.22×10^− 4^ | 0.05 | 0.36 | 1.81 |
|  | Hexanoylcarnitine (Car(6:0)) | C_13_H_25_NO_4_ | 260.188 | 140.4 | 0.04 | 0.29 | 0.62 | 1.77 |
|  | Car(18:1) | C_25_H_47_NO_4_ | 426.3619 | 94.9 | 2.97×10^− 3^ | 0.08 | 0.84 | 1.76 |
|  | Prolylhydroxyproline | C_10_H_16_N_2_O_4_ | 229.1204 | 245.9 | 0.02 | 0.20 | 0.31 | 1.74 |
|  | N1,N8-Diacetylspermidine | C_11_H_23_N_3_O_2_ | 230.1884 | 198.8 | 5.24×10^− 4^ | 0.04 | 0.28 | 1.82 |
|  | Propionylcarnitine (Car(3:0)) | C_10_H_19_NO_4_ | 218.1407 | 170.4 | 1.08×10^− 4^ | 0.03 | 3.09 | 1.82 |
|  | Valerylcarnitine | C_12_H_23_NO_4_ | 246.1723 | 149.6 | 4.71×10^− 4^ | 0.04 | 1.81 | 1.83 |
|  | 1-O-Hexadecyl-sn-glycero-3-phosphocholine (LPC(O-16:0/0:0)) | C_24_H_52_NO_6_P | 482.3647 | 125.9 | 5.55×10^− 4^ | 0.04 | 0.62 | 1.81 |
|  | 1-Myristoyl-sn-glycero-3-phosphocholine (LPC(14:0/0:0)) | C_22_H_46_NO_7_P | 468.3128 | 125.4 | 1.95×10^− 3^ | 0.07 | 0.60 | 1.80 |
|  | Acetylcholine | C_7_H_16_NO_2_ | 146.1188 | 105.7 | 0.01 | 0.10 | 0.50 | 1.85 |
|  | Melibiose | C_12_H_22_O_11_ | 365.1089 | 231.1 | 0.01 | 0.17 | 0.22 | 1.83 |
|  | Isovalerylcarnitine (Car(5:0)) | C_12_H_23_NO_4_ | 246.1723 | 149.6 | 4.71×10^− 4^ | 0.04 | 1.81 | 1.83 |
|  | Pyrrolidine | C_4_H_9_N | 72.0814 | 180.3 | 0.02 | 0.22 | 0.16 | 1.47 |
|  | Mannose | C_6_H_12_O_6_ | 179.0568 | 176.1 | 0.04 | 0.30 | 0.28 | 1.80 |
|  | Turanose | C_12_H_22_O_11_ | 341.1105 | 230.2 | 0.03 | 0.23 | 0.42 | 1.81 |
|  | 3-Methyluridine | C_10_H_14_N_2_O_6_ | 257.0789 | 54.4 | 4.47×10^− 4^ | 0.04 | 0.43 | 1.82 |
|  | Sucrose | C_12_H_22_O_11_ | 341.1105 | 230.2 | 0.03 | 0.23 | 0.42 | 1.81 |
|  | Car(18:0) | C_25_H_49_NO_4_ | 428.3779 | 93.7 | 0.04 | 0.29 | 0.73 | 1.54 |
|  | 4-(Methylamino)butanoic acid | C_5_H_11_NO_2_ | 116.0721 | 180.4 | 0.04 | 0.30 | 0.44 | 1.77 |
|  | 1-Methylpseudouridine | C_10_H_14_N_2_O_6_ | 257.0789 | 54.4 | 4.47×10^− 4^ | 0.04 | 0.43 | 1.82 |
|  | Fructose | C_6_H_12_O_6_ | 179.0568 | 176.1 | 0.04 | 0.30 | 0.28 | 1.80 |
|  | Adenosine 3'-monophosphate | C_10_H_14_N_5_O_7_P | 348.0738 | 250.7 | 0.02 | 0.18 | 0.59 | 1.70 |
|  | L-Azetidine-2-carboxylic acid | C_4_H_7_NO_2_ | 102.0558 | 232 | 6.28×10^− 4^ | 0.04 | 0.36 | 1.84 |
|  | Caplamin | C_6_H_13_NO_2_ | 130.0878 | 163.5 | 0.01 | 0.10 | 0.40 | 1.78 |
|  | 2-Aminoisobutyric acid | C_4_H_9_NO_2_ | 102.0564 | 231.7 | 2.28×10^− 3^ | 0.07 | 0.44 | 1.79 |
|  | LPC(18:0/0:0) | C_26_H_54_NO_7_P | 524.3755 | 119.4 | 1.44×10^− 3^ | 0.06 | 0.64 | 1.78 |
|  | Palmitoylcarnitine (Car(16:0)) | C_23_H_45_NO_4_ | 400.3458 | 99.4 | 0.03 | 0.24 | 0.78 | 1.72 |
|  | 2,4-Dihydroxybutanoic acid | C_4_H_8_O_4_ | 101.0247 | 116.4 | 0.01 | 0.12 | 0.30 | 1.66 |
|  | N-Methylglutamic acid | C_6_H_11_NO_4_ | 160.0622 | 237.6 | 2.02×10^− 3^ | 0.07 | 0.70 | 1.78 |
|  | N-Acetylglutamic acid | C_7_H_11_NO_5_ | 188.0572 | 225.3 | 0.01 | 0.11 | 0.64 | 1.75 |
|  | Galactinol | C_12_H_22_O_11_ | 341.1105 | 230.2 | 0.03 | 0.23 | 0.42 | 1.81 |
|  | o-Tyrosine | C_9_H_11_NO_3_ | 180.0673 | 182.6 | 0.01 | 0.13 | 0.34 | 1.77 |
|  | 4,4'-Sulfonylbisphenol | C_12_H_10_O_4_S | 249.0229 | 175.1 | 6.29×10^− 4^ | 0.04 | 0.37 | 1.79 |
|  | 2,3-Bis(4-fluorophenyl)-6-quinoxalinecarboxylic acid | C_21_H_12_F_2_N_2_O_2_ | 363.0933 | 175.3 | 8.87×10^− 4^ | 0.05 | 0.30 | 1.74 |
|  | Nivalenol | C_15_H_20_O_7_ | 335.1092 | 176.1 | 1.75×10^− 4^ | 0.03 | 0.14 | 1.84 |
|  | 5,7,3',4',5'-Pentahydroxyflavone | C_15_H_10_O_7_ | 301.0346 | 252 | 0.01 | 0.15 | 0.58 | 1.73 |
|  | 4-(Pentafluorosulfanyl)aniline | C_6_H_6_F_5_NS | 220.0221 | 232.5 | 0.02 | 0.21 | 1.37 | 1.65 |
|  | (S)-5-Methylhydantoin | C_4_H_6_N_2_O_2_ | 113.036 | 172.4 | 4.04×10^− 4^ | 0.04 | 0.49 | 1.82 |
|  | 4-Fluorobenzenesulfonamide | C_6_H_6_FNO_2_S | 174.0026 | 124.8 | 3.48×10^− 3^ | 0.08 | 0.53 | 1.85 |
|  | Gramine | C_11_H_14_N_2_ | 175.1205 | 293.9 | 0.03 | 0.27 | 0.74 | 1.77 |
|  | Methyl 9H-purine-6-carboxylate | C_7_H_6_N_4_O_2_ | 177.0411 | 58.7 | 0.04 | 0.29 | 0.60 | 1.50 |
|  | (Z)-6-Octadecenoic acid | C_18_H_34_O_2_ | 281.2495 | 19.4 | 0.04 | 0.31 | 1.10 | 1.52 |
|  | Tolmetin_sodium | C_15_H_14_NNaO_3_ | 280.0946 | 226 | 0.03 | 0.23 | 0.63 | 1.57 |
|  | Isobutyric_acid | C_4_H_8_O_2_ | 87.0454 | 50.5 | 0.01 | 0.10 | 0.66 | 1.74 |
|  | Penicillin G | C_16_H_18_N_2_O_4_S | 333.0926 | 39.9 | 0.02 | 0.19 | 0.23 | 1.83 |
|  | 2-Hydroxymyristic_Acid | C_14_H_28_O_3_ | 243.1975 | 29.9 | 3.25×10^− 4^ | 0.04 | 0.70 | 1.84 |
|  | Succinic semialdehyde | C_4_H_6_O_3_ | 101.0248 | 216.7 | 0.01 | 0.16 | 0.51 | 1.82 |
|  | 4-(4-Nitrophenyl)-1H-imidazole | C_9_H_7_N_3_O_2_ | 188.0459 | 125.7 | 2.21×10^− 3^ | 0.07 | 0.16 | 1.79 |
|  | Pterine | C_6_H_5_N_5_O | 162.0427 | 95.1 | 0.02 | 0.20 | 0.75 | 1.61 |
|  | N-Methyl-N-(methylsulfonyl)glycine | C_4_H_9_NO_4_S | 166.0186 | 77.6 | 1.16×10^− 3^ | 0.05 | 0.24 | 1.83 |
|  | L-Menthone_1,2-glycerol_ketal | C_16_H_15_N_3_OS | 298.0998 | 275.7 | 0.02 | 0.19 | 0.68 | 1.59 |
|  | 7-Chloro-5-methyl-2H-1,4-benzothiazin-3(4H)-one | C_9_H_8_ClNOS | 214.0083 | 232.5 | 0.01 | 0.16 | 0.51 | 1.83 |
|  | 5-(2-methoxyethylamino)-3H-1,3,4-thiadiazole-2-thione | C_5_H_9_N_3_OS_2_ | 192.0261 | 232 | 8.84×10^− 4^ | 0.05 | 0.40 | 1.84 |
|  | Bicyclo-prostaglandin E1 | C_20_H_32_O_4_ | 335.2216 | 22.5 | 0.03 | 0.23 | 1.34 | 1.59 |
|  | 2,2'-Iminodiacetic acid | C_4_H_7_NO_4_ | 132.0308 | 256.9 | 3.06×10^− 3^ | 0.08 | 1.51 | 1.77 |
|  | 5'-Phosphoribosyl-5-amino-4-imidazolecarboxamide (AICAR) | C_9_H_15_N_4_O_8_P | 319.0452 | 252.1 | 1.42×10^− 3^ | 0.06 | 0.64 | 1.81 |
|  | Isoleucylisoleucine | C_12_H_24_N_2_O_3_ | 245.1881 | 100.4 | 0.02 | 0.22 | 0.55 | 1.65 |
|  | Thr-Val | C_9_H_18_N_2_O_4_ | 217.1201 | 161.2 | 0.05 | 0.33 | 0.43 | 1.75 |
|  | Alnustone | C_19_H_18_O | 263.1447 | 102 | 0.02 | 0.21 | 0.50 | 1.67 |
|  | 2-(4-Phenyl-1-piperidinyl)cyclohexanol | C_17_H_25_NO | 260.1993 | 296.1 | 1.23×10^− 3^ | 0.06 | 0.60 | 1.82 |
|  | Platencin | C_24_H_27_NO_6_ | 424.1793 | 14.2 | 0.02 | 0.22 | 0.56 | 1.56 |
|  | .alpha.-Pyrrolidinohexanophenone | C_16_H_23_NO | 246.1835 | 301 | 3.79×10^− 3^ | 0.08 | 0.65 | 1.85 |
|  | 1H-3-Benzazepine-7,8-diol, 6-chloro-2,3,4,5-tetrahydro-1-phenyl- | C_16_H_16_ClNO_2_ | 288.0776 | 164.3 | 0.05 | 0.33 | 0.35 | 1.77 |
|  | Aristolodione | C_18_H_13_NO_4_ | 308.0941 | 246.1 | 0.01 | 0.14 | 0.74 | 1.74 |
|  | 3-Amino-4-hydroxyphenyl sulfone | C_12_H_12_N_2_O_4_S | 281.057 | 168.5 | 1.38×10^− 4^ | 0.03 | 0.52 | 1.84 |
|  | 1-Deoxy-D-glucitol | C_6_H_14_O_5_ | 167.091 | 158.6 | 1.05×10^− 3^ | 0.05 | 0.42 | 1.83 |
|  | Zederone | C_15_H_18_O_3_ | 247.131 | 50.1 | 1.94×10^− 3^ | 0.07 | 0.27 | 1.82 |
|  | Isoleucylvaline | C_11_H_22_N_2_O_3_ | 231.1723 | 113.6 | 0.04 | 0.30 | 0.51 | 1.63 |
|  | 6-Chloro-7-fluoroisatin | C_8_H_3_ClFNO_2_ | 199.9925 | 238.6 | 0.01 | 0.14 | 1.77 | 1.65 |
|  | N-(4-Hydroxyphenyl)propanamide | C_9_H_11_NO_2_ | 166.0877 | 158.6 | 8.22×10^− 4^ | 0.05 | 0.43 | 1.84 |
|  | 1H-Benzimidazole-2-carboxylic acid | C_8_H_6_N_2_O_2_ | 163.0516 | 125.1 | 0.05 | 0.33 | 0.52 | 1.77 |
|  | 2-Cyclohexyl-N-phenylacetamide | C_14_H_19_NO | 218.1519 | 251.8 | 0.04 | 0.28 | 0.40 | 1.58 |
|  | 2-(2,6-Dimethylmorpholin-4-yl)ethanol | C_8_H_17_NO_2_ | 160.1346 | 226 | 7.13×10^− 4^ | 0.05 | 0.67 | 1.82 |
|  | Costunolide | C_15_H_20_O_2_ | 233.1517 | 150.2 | 0.01 | 0.17 | 0.46 | 1.72 |
|  | 2-Pyrimidinylmethanol | C_5_H_6_N_2_O | 111.0563 | 143.8 | 3.21×10^− 3^ | 0.08 | 0.60 | 1.80 |
|  | 3-(4-Fluorophenyl)-2-thioxo-2,3-dihydro-4(1H)-quinazolinone | C_14_H_9_FN_2_OS | 273.0508 | 131.6 | 0.02 | 0.17 | 0.62 | 1.73 |
|  | Methyl 6-hydroxy-1H-indole-3-carboxylate | C_10_H_9_NO_3_ | 190.0526 | 125.7 | 0.05 | 0.32 | 0.58 | 1.52 |
|  | 1-O-Octadecyl-sn-glyceryl-3-phosphorylcholine | C_26_H_56_NO_6_P | 510.3961 | 122.9 | 3.60×10^− 4^ | 0.04 | 0.67 | 1.82 |
|  | 2-Bromoestra-1,3,5(10)-triene-3,17-diol | C_18_H_23_BrO_2_ | 349.0777 | 120.6 | 2.64×10^− 4^ | 0.04 | 1.35 | 1.82 |
|  | Miltefosine | C_21_H_46_NO_4_P | 408.327 | 110.2 | 0.03 | 0.24 | 0.74 | 1.64 |
|  | LPC(O-18:1) | C_26_H_54_NO_6_P | 508.3804 | 123.7 | 0.01 | 0.13 | 0.69 | 1.69 |
|  | LPC(18:1) | C_26_H_52_NO_7_P | 522.3599 | 119.5 | 4.85×10^− 3^ | 0.09 | 0.65 | 1.71 |
|  | PC(38:6) | C_46_H_80_NO_8_P | 788.5665 | 30.2 | 4.09×10^− 3^ | 0.09 | 1.43 | 1.76 |
|  | 4-Fluoro-N-phenylbenzenesulfonamide | C_12_H_10_FNO_2_S | 250.032 | 232 | 8.89×10^− 4^ | 0.05 | 0.47 | 1.83 |
|  | imidazo[1,2-c]pyrimidin-5-ol | C_6_H_5_N_3_O | 136.0492 | 205.5 | 3.60×10^− 3^ | 0.08 | 0.64 | 1.79 |
|  | N-(4-Methyl-1,3-thiazol-2-yl)-N-(1-naphthyl)amine | C_14_H_12_N_2_S | 241.0817 | 179.8 | 0.01 | 0.11 | 0.19 | 1.76 |
|  | 2-(4-(Methylamino)phenyl)benzo[d]thiazol-6-ol | C_14_H_12_N_2_OS | 257.0767 | 131.8 | 0.02 | 0.22 | 0.65 | 1.70 |
|  | Glycerophospho-N-palmitoylethanolamine | C_21_H_44_NO_7_P | 454.2971 | 125.1 | 0.01 | 0.17 | 0.87 | 1.65 |
|  | 1-(1Z-Octadecenyl)-sn-glycero-3-phosphocholine | C_26_H_54_NO_6_P | 508.3805 | 108.8 | 0.01 | 0.14 | 0.81 | 1.69 |
|  | Arg-Ser | C_9_H_19_N_5_O_4_ | 262.1535 | 249.4 | 0.03 | 0.24 | 0.27 | 1.55 |
|  | Pro-Val | C_10_H_18_N_2_O_3_ | 215.141 | 177.9 | 4.66×10^− 3^ | 0.09 | 0.62 | 1.72 |
|  | Phthalimide | C_8_H_5_NO_2_ | 148.0406 | 153.2 | 0.02 | 0.21 | 0.31 | 1.03 |
|  | Lyso-sphingomyelin | C_23_H_49_N_2_O_5_P | 465.3497 | 132.8 | 0.02 | 0.20 | 0.85 | 1.67 |
|  | 1,3-Diphenyl-2-propanone | C_15_H_14_O | 211.1096 | 49.9 | 6.88×10^− 4^ | 0.04 | 0.27 | 1.82 |
|  | Heptenoylcarnitine (Car(7:1)) | C_14_H_25_NO_4_ | 272.1882 | 148.3 | 0.01 | 0.11 | 0.68 | 1.76 |
|  | 1-(2-Oxo-2-phenylethyl)pyridinium cation | C_13_H_12_NO | 198.0892 | 172.9 | 0.01 | 0.13 | 0.12 | 1.84 |
|  | 5-Hydroxy-6E,8Z,11Z,14Z-eicosatetraenoic acid, 1,5-lactone | C_20_H_30_O_2_ | 303.2346 | 17.1 | 0.01 | 0.14 | 2.13 | 1.67 |
|  | Gallic_acid | C_7_H_6_O_5_ | 169.0124 | 51.1 | 0.01 | 0.12 | 0.49 | 1.69 |
|  | Amprolium cation | C_14_H_19_N_4_ | 243.1599 | 303.9 | 0.01 | 0.11 | 0.80 | 1.74 |
|  | Thieno[2,3-b]pyridine-5-carbonitrile, 6,7-dihydro-4-hydroxy-3-(2'-hydroxy[1,1'-biphenyl]-4-yl)-6-oxo- | C_20_H_12_N_2_O_3_S | 359.0461 | 231.7 | 3.87×10^− 4^ | 0.04 | 0.20 | 1.84 |
|  | 6,6'-Dihydroxy-5,5'-dimethoxybiphenyl-3,3'-dicarboxylic acid | C_16_H_14_O_8_ | 333.0609 | 251 | 0.01 | 0.10 | 0.62 | 1.76 |
|  | Pro-Pro | C_10_H_16_N_2_O_3_ | 213.1254 | 241.3 | 1.57×10^− 3^ | 0.06 | 0.66 | 1.81 |
|  | 3-Phosphonopropanoic acid | C_3_H_7_O_5_P | 152.9963 | 160.3 | 0.03 | 0.26 | 0.49 | 1.78 |
|  | Isopentenyladenine | C_10_H_13_N_5_ | 202.1093 | 88.2 | 0.02 | 0.22 | 0.13 | 1.84 |
|  | Mukoline | C_14_H_13_NO_2_ | 228.1 | 182.3 | 1.58×10^− 3^ | 0.06 | 0.43 | 1.82 |
|  | (R)-(-)-2-Oxothiazolidine-4-carboxylic acid | C_4_H_5_NO_3_S | 148.0052 | 175.3 | 0.02 | 0.18 | 0.62 | 1.67 |
|  | Entacapone | C_14_H_15_N_3_O_5_ | 304.0975 | 103.1 | 0.03 | 0.23 | 0.40 | 1.80 |
|  | 2-Butylquinoline | C_13_H_15_N | 186.1253 | 302.9 | 3.84×10^− 3^ | 0.09 | 0.81 | 1.78 |
|  | 2-{Bis[4-(dimethylamino)phenyl]methyl}phenol | C_23_H_26_N_2_O | 347.2072 | 249.6 | 0.01 | 0.15 | 0.35 | 1.73 |
|  | 2-sulfanylthieno[3,2-d]pyrimidin-4(1H)-one | C_6_H_4_N_2_OS_2_ | 184.9814 | 224.6 | 2.41×10^− 4^ | 0.04 | 0.36 | 1.85 |
|  | Cyclopropanecarboxamide, N-[3-[[6-[[3-(trifluoromethyl)phenyl]amino]-4-pyrimidinyl]amino]phenyl]- | C_21_H_18_F_3_N_5_O | 414.1594 | 278.6 | 0.01 | 0.15 | 0.64 | 1.65 |
|  | N-(4-Chloro-3-nitrophenyl)-3-methylbenzamide | C_14_H_11_ClN_2_O_3_ | 289.0345 | 267.4 | 0.03 | 0.23 | 0.75 | 1.61 |
|  | 3-(4-Bromophenyl)-7-hydroxy-4H-chromen-4-one | C_15_H_9_BrO_3_ | 314.9671 | 247 | 0.02 | 0.21 | 0.44 | 1.81 |
|  | N-3,4-Tridhydroxybenzamide | C_7_H_7_NO_4_ | 168.0285 | 231.7 | 3.88×10^− 4^ | 0.04 | 0.40 | 1.83 |
|  | 3-Chloro-2-hydroxy-5-phenylbenzoic acid | C_13_H_9_ClO_3_ | 247.0144 | 124 | 0.02 | 0.23 | 0.64 | 1.66 |
|  | risedronate | C_7_H_11_NO_7_P_2_ | 281.9977 | 175.3 | 0.03 | 0.25 | 0.64 | 1.64 |
|  | 6-Fluoro-2-(pyridin-2-yl)-1H-1,3-benzodiazole | C_12_H_8_FN_3_ | 212.066 | 205.4 | 5.75×10^− 4^ | 0.04 | 0.64 | 1.81 |
|  | 3-Hydroxyisovalerylcarnitine | C_12_H_23_O_5_N | 262.1673 | 183.6 | 1.29×10^− 3^ | 0.06 | 0.71 | 1.82 |
|  | 2,2,4-Trimethyl-1,2,3,4-tetrahydroquinoline | C_12_H_17_N | 176.1408 | 165.6 | 0.04 | 0.30 | 0.78 | 1.56 |
|  | 3-[4-Fluoro-3-(trifluoromethyl)phenyl]-5,5-dimethyl-hydantoin | C_12_H_10_F_4_N_2_O_2_ | 289.0564 | 120.6 | 2.85×10^− 4^ | 0.04 | 1.36 | 1.81 |
|  | Hydroxyprolyl-Proline | C_10_H_16_N_2_O_4_ | 229.1203 | 50 | 2.59×10^− 3^ | 0.07 | 0.26 | 1.82 |
|  | 2-Chloro-N-(4-methoxyphenyl)nicotinamide | C_13_H_11_ClN_2_O_2_ | 263.0551 | 280.7 | 0.03 | 0.25 | 0.78 | 1.59 |
|  | N,N-Diisopropyl 4-(4-Bromopyrazol-1-yl)benzenesulfonamide | C_15_H_20_BrN_3_O_2_S | 386.0512 | 267.5 | 0.03 | 0.23 | 0.68 | 1.63 |
|  | 1,3-Dihydroxy-7,8,9,10-tetrahydro-6H-benzo[c]chromen-6-one | C_13_H_12_O_4_ | 231.0632 | 229.3 | 3.48×10^− 3^ | 0.08 | 0.68 | 1.77 |
|  | Tricin methyl ether | C_18_H_16_O_7_ | 343.0782 | 231.7 | 9.91×10^− 4^ | 0.05 | 0.44 | 1.82 |
|  | L-isoleucyl-L-proline | C_11_H_20_N_2_O_3_ | 229.1568 | 181.3 | 0.01 | 0.13 | 0.62 | 1.84 |
|  | Pisumionoside | C_19_H_32_O_9_ | 405.2133 | 233 | 3.59×10^− 3^ | 0.08 | 0.40 | 1.79 |
|  | Pyrovalerone | C_16_H_23_NO | 246.1835 | 312.6 | 2.52×10^− 4^ | 0.04 | 0.42 | 1.83 |
|  | Glu-Ala | C_8_H_14_N_2_O_5_ | 217.0838 | 179.9 | 1.59×10^− 3^ | 0.06 | 0.27 | 1.83 |
|  | Amino(4-methoxyphenyl)acetic acid | C_9_H_11_NO_3_ | 182.0801 | 242.9 | 0.02 | 0.21 | 1.37 | 1.58 |
|  | Isopropalin | C_15_H_23_N_3_O_4_ | 310.1792 | 248.6 | 4.06×10^− 3^ | 0.09 | 0.63 | 1.79 |
|  | Thioridazine | C_21_H_26_N_2_S_2_ | 371.1665 | 14.6 | 0.02 | 0.19 | 0.68 | 1.64 |
|  | Pro-Leu | C_11_H_20_N_2_O_3_ | 229.1567 | 217.5 | 1.03×10^− 3^ | 0.05 | 0.66 | 1.82 |
|  | Dynasore | C_18_H_14_N_2_O_4_ | 321.089 | 244.4 | 3.28×10^− 3^ | 0.08 | 0.81 | 1.77 |
|  | [3-(Trifluoromethyl)-1H-pyrazol-1-yl]acetic acid | C_6_H_5_F_3_N_2_O_2_ | 193.0236 | 34.2 | 0.01 | 0.12 | 0.66 | 1.69 |
|  | LysoPC(18:3(6Z,9Z,12Z)) | C_26_H_48_NO_7_P | 518.3263 | 121.5 | 3.63×10^− 3^ | 0.08 | 0.67 | 1.78 |
|  | 2-Methoxy-N-(1H-tetraazol-5-yl)benzamide | C_9_H_9_N_5_O_2_ | 218.0679 | 221.3 | 4.37×10^− 3^ | 0.09 | 0.67 | 1.78 |
|  | 1-[(4-Nitrophenyl)sulfonyl]piperidine-4-carboxylic acid | C_12_H_14_N_2_O_6_S | 313.0459 | 256.4 | 0.02 | 0.22 | 0.72 | 1.63 |
|  | Heptanedioic acid, 1-(2-cyclopentylidenehydrazide) | C_12_H_20_N_2_O_3_ | 241.1569 | 219.9 | 0.02 | 0.21 | 0.75 | 1.79 |
|  | PC(P-18:1(9Z)/0:0) | C_26_H_52_NO_6_P | 506.3649 | 110.6 | 0.01 | 0.17 | 0.80 | 1.64 |
|  | .beta.-D-Glucopyranosiduronic acid, 4-(7-hydroxy-4-oxo-4H-1-benzopyran-3-yl)phenyl | C_21_H_18_O_10_ | 429.0817 | 18.1 | 0.02 | 0.20 | 0.60 | 1.66 |
|  | 4,5-Dihydro-2-methylthiazole | C_4_H_7_NS | 102.0381 | 172.9 | 0.02 | 0.18 | 0.58 | 1.82 |
|  | ACARBOSE | C_25_H_43_NO_18_ | 684.2095 | 16.2 | 0.03 | 0.25 | 1.15 | 1.62 |
|  | .beta.-Glutamic acid | C_5_H_9_NO_4_ | 148.0617 | 232 | 4.98×10^− 4^ | 0.04 | 0.33 | 1.84 |
|  | 2-[2-(3,4-Dihydroxyphenyl)-2-oxoethyl]thio-4-(2-furyl)-6-methylnicotinonitrile | C_19_H_14_N_2_O_4_S | 365.0601 | 231.8 | 1.47×10^− 3^ | 0.06 | 0.53 | 1.81 |
|  | 1-Trifluoromethoxyphenyl-3-(1-propionylpiperidin-4-yl)urea | C_16_H_20_F_3_N_3_O_3_ | 360.1537 | 230.6 | 0.01 | 0.10 | 0.35 | 1.85 |
|  | Pyrrocidine B | C_31_H_39_NO_4_ | 490.2951 | 125.5 | 2.57×10^− 3^ | 0.07 | 0.60 | 1.73 |
|  | LysoPC(22:1(13Z)) | C_30_H_60_NO_7_P | 578.4232 | 114.4 | 4.85×10^− 3^ | 0.09 | 0.76 | 1.73 |
|  | N-Methyl-D-aspartic acid | C_5_H_9_NO_4_ | 148.0617 | 284.8 | 0.01 | 0.17 | 0.54 | 1.62 |
|  | 4-Bromo-2-{[4-(2-pyridinylmethyl)-1-piperazinyl]methyl}phenol | C_17_H_20_BrN_3_O | 362.0872 | 226 | 4.00×10^− 3^ | 0.09 | 0.83 | 1.75 |
|  | Xerocomic_acid | C_18_H_12_O_8_ | 357.0592 | 250.7 | 0.01 | 0.11 | 0.69 | 1.75 |
|  | PC(16:1(9Z)/0:0)[U] | C_24_H_48_NO_7_P | 494.3286 | 122.4 | 0.01 | 0.14 | 0.73 | 1.69 |
|  | N,N-Diisopropyl-4-bromo-3-methoxybenzamide | C_14_H_20_BrNO_2_ | 314.0778 | 274.8 | 3.22×10^− 3^ | 0.08 | 0.53 | 1.77 |
|  | Cedrin | C_16_H_14_O_8_ | 335.077 | 250.7 | 3.33×10^− 3^ | 0.08 | 0.65 | 1.79 |
|  | Primaquine | C_15_H_21_N_3_O | 260.1742 | 238.6 | 0.01 | 0.11 | 0.47 | 1.75 |
|  | S-(4-Fluorophenyl)mercapturic acid | C_11_H_12_FNO_3_S | 256.0458 | 232.1 | 0.03 | 0.23 | 1.30 | 1.63 |
|  | Erythronolactone | C_4_H_6_O_4_ | 117.0198 | 224.3 | 0.02 | 0.20 | 0.33 | 1.82 |
|  | 2-{[(2-Oxo-2H-chromen-3-yl)carbonyl]amino}benzoic acid | C_17_H_11_NO_5_ | 308.0574 | 240.2 | 4.37×10^− 3^ | 0.09 | 0.70 | 1.78 |
|  | Thr-Asp | C_8_H_14_N_2_O_6_ | 233.0787 | 131.4 | 0.04 | 0.29 | 0.75 | 1.58 |
|  | Nitrofurazone | C_6_H_6_N_4_O_4_ | 197.0323 | 53.9 | 0.03 | 0.24 | 0.78 | 1.63 |
|  | N-Nitroso-N-methyl-3-aminopropionic acid, methyl ester | C_5_H_10_N_2_O_3_ | 147.0777 | 247.1 | 0.01 | 0.14 | 0.33 | 1.73 |
|  | Succinamide | C_4_H_8_N_2_O_2_ | 117.0668 | 214.9 | 1.06×10^− 3^ | 0.05 | 0.40 | 1.83 |
|  | 4-Phenyl-2-sulfanyl-3,4-dihydro-5H-indeno[1,2-d]pyrimidin-5-one | C_17_H_12_N_2_OS | 291.0583 | 125.2 | 0.04 | 0.29 | 0.08 | 1.80 |
|  | Eupatorin | C_18_H_16_O_7_ | 367.0768 | 232 | 0.02 | 0.19 | 0.56 | 1.82 |
|  | Fenoxaprop-P | C_16_H_12_ClNO_5_ | 332.0356 | 231.8 | 1.11×10^− 3^ | 0.05 | 0.51 | 1.82 |
|  | Carprofen | C_15_H_12_ClNO_2_ | 272.0464 | 183.7 | 0.04 | 0.29 | 0.45 | 1.78 |
|  | 1,7-Dioxa-4,10-diazacyclododecane | C_8_H_18_N_2_O_2_ | 175.1455 | 305.4 | 0.01 | 0.11 | 0.73 | 1.73 |
|  | Labienoxime | C_13_H_23_NO | 210.1833 | 356.5 | 0.03 | 0.26 | 0.87 | 1.61 |
|  | 1-[(2-Nitrophenyl)sulfonyl]piperidine-2-carboxylic acid | C_12_H_14_N_2_O_6_S | 315.0619 | 255.8 | 0.01 | 0.14 | 0.73 | 1.72 |
|  | 2-Chloro-6-methylbenzoic acid | C_8_H_7_ClO_2_ | 169.0078 | 74.2 | 0.04 | 0.28 | 0.73 | 1.62 |
|  | 6-Bromo-2-[3-(2-methylpropoxy)phenyl]quinoline-4-carboxylic acid | C_20_H_18_BrNO_3_ | 398.0357 | 87.7 | 1.73×10^− 3^ | 0.06 | 0.29 | 1.82 |
|  | Ethyl 7-chloro-1-(2,4-difluorophenyl)-6-fluoro-4-oxo-1,4-dihydro[1,8]naphthyridine-3-carboxylate | C_17_H_10_ClF_3_N_2_O_3_ | 383.0449 | 232.1 | 8.49×10^− 4^ | 0.05 | 0.23 | 1.84 |
|  | 4-Acetyloxy-8-(3-oxo-2-pent-2-enylcyclopenten-1-yl)octanoic acid | C_20_H_30_O_5_ | 349.2067 | 22.8 | 0.04 | 0.31 | 1.36 | 1.51 |
|  | 1-(4-Bromophenylsulfonyl)-4-butylpiperazine | C_14_H_21_BrN_2_O_2_S | 361.063 | 232 | 8.88×10^− 4^ | 0.05 | 0.22 | 1.84 |
|  | 2-[(Methylsulfonyl)anilino]propanoic acid | C_10_H_13_NO_4_S | 242.0527 | 175.4 | 0.01 | 0.12 | 0.11 | 1.77 |
|  | glutathione sulfinate | C_10_H_17_N_3_O_8_S | 340.0843 | 264.2 | 0.05 | 0.33 | 0.80 | 1.54 |
|  | N-Gluconyl ethanolamine phosphate | C_8_H_18_NO_10_P | 318.0611 | 235.4 | 2.36×10^− 3^ | 0.07 | 0.70 | 1.79 |
|  | 2-Aminoadenosine | C_10_H_14_N_6_O_4_ | 281.1001 | 161 | 1.37×10^− 4^ | 0.03 | 0.23 | 1.84 |
|  | Aspartylhydroxyproline | C_9_H_14_N_2_O_6_ | 247.0961 | 255.8 | 0.04 | 0.30 | 0.43 | 1.62 |
|  | M361T237 | C_13_H_24_N_6_O_6_ | 361.1867 | 236.6 | 0.01 | 0.15 | 0.20 | 1.84 |
|  | N-acetylglycine | C_4_H_7_NO_3_ | 116.0357 | 188.8 | 0.04 | 0.29 | 0.60 | 1.76 |
|  | 1-(9Z-Nonadecenoyl)-glycero-3-phosphoethanolamine | C_24_H_48_NO_7_P | 492.3113 | 116.4 | 0.05 | 0.32 | 0.39 | 1.66 |
|  | M306T232 | C_11_H_15_NO_9_ | 306.0784 | 232.5 | 0.04 | 0.29 | 0.75 | 1.76 |
|  | 3,4-dihydroxypent-3-en-2-one 5-phosphate | C_5_H_9_O_7_P | 211.0022 | 171.9 | 1.61×10^− 3^ | 0.06 | 0.69 | 1.79 |
